# Genetic Diversity and Distribution of Human Norovirus in China (1999–2011)

**DOI:** 10.1155/2014/196169

**Published:** 2014-01-21

**Authors:** Yongxin Yu, Shuling Yan, Bailin Li, Yingjie Pan, Yongjie Wang

**Affiliations:** ^1^Shanghai Engineering Research Center of Aquatic-Product Processing & Preservation, Shanghai 201306, China; ^2^College of Food Science and Technology, Shanghai Ocean University, Shanghai 201306, China; ^3^Institute of Biochemistry and Molecular Cell Biology, University of Goettingen, 37077 Goettingen, Germany

## Abstract

Noroviruses (NoVs) are a leading cause of epidemic and sporadic acute gastroenteritis worldwide. However, the genetic diversity and geographical distribution of NoV isolates from China have not been well described thus far. In this study, all NoV sequences obtained in China from 1999 to 2011 (*n* = 983), both partial and complete genomes, were downloaded from GenBank. Genotyping and phylogenetic and recombination analyses were performed in order to gain a better understanding of the distribution and genetic diversity of NoVs in China. The results indicated that approximately 90% of NoV sequences were obtained from the coastal regions of China, and most of the NoV sequences from distinct geographical regions appeared to be closely related. GII.4 was the most prevalent genotype, accounting for 64.4% of all genotypes, followed by GII.12 (13.9%) and GII.3 (7.0%). Over the last decade, the GII.4 variants were dominated by successive circulation of GII.4/2002, GII.4/2004, GII.4/2006b, and GII.4/2008, with GII.4/2006b continuing to date. A relatively high frequency of NoV intergenotype recombinants was identified. The most common ORF1/ORF2 intergenotype recombinant was GII.12/GII.4 (*n* = 11), and the relative frequency was up to 30% among all the recombinant strains (*n* = 36). These findings may aid in the evaluation and implementation of appropriate measures for monitoring NoV infectious diseases in China.

## 1. Introduction

Norovirus (NoV) is currently considered to be the most common cause of nonbacterial gastroenteritis outbreaks in both developed and developing countries [1–4]. People of all ages can be infected by this virus. NoV related outbreaks are most often reported in closed settings [5–8], for example, schools, cruise ships, and hospitals, where infections in high-risk groups, such as the elderly and the immunocompromised, can have a serious impact by causing prolonged morbidity and mortality [9, 10].

**Figure 1:**
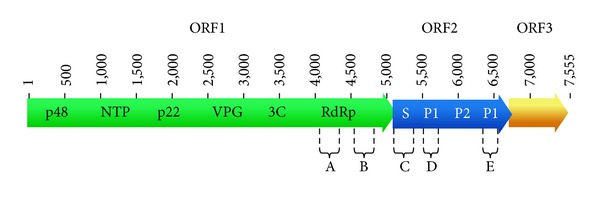
Genome map of norovirus (NoV; X86557). The NoV genome is composed of 3 open reading frames (ORFs). ORF1 (~5 kb) encodes the nonstructural proteins of an NTPase, 3C-like protease, and RNA-dependent RNA polymerase (RdRp). ORF2 is 1.8 kb in length and encodes the 57 kDa major structural capsid protein of viral protein 1 (VP1). VP1 is divided into the shell domain (S) and the protruding domain (P). The P domain contains two subdomains, known as P1 and P2. The regions of A, B, C, D, and E on NoV genome are used for genotyping. ORF3 encodes a minor structural protein VP2.

NoV is a small round virion of 27–38 nm in diameter and contains a single-stranded, positive-sense, and polyadenylated RNA genome of 7400–7700 nucleotides [11]. The NoV genome contains 3 open reading frames (ORFs) [2, 12] (Figure 1). ORF1 encodes for the nonstructural proteins, including an NTPase, 3C-like protease, and RNA-dependent RNA polymerase (RdRp) [9]. ORF2 encodes the major structural protein (VP1) that forms the capsid, and ORF3 encodes a minor structural protein VP2 [13]. The VP1 consists of 3 domains, the shell (S), P1, and P2. The S domain is responsible for assembly of VP1, and the P1 domain enhances the stability of the virus particles [14]. The viral capsid contains 180 copies of the VP1 protein and a few copies of VP2, and in vitro expression of the VP1 gene leads to the spontaneous formation of virus-like particles (VLPs) [15].

*Norovirus* is a highly diverse genus in the* Caliciviridae* family [2, 11, 16]. Presently, it comprises 5 distinct genogroups, GI, GII, GIII, GIV, and GV, on the basis of sequence analysis of VP1. Of these, GI, GII, and GIV strains have been detected in humans [11]. These 3 genogroups can be further subdivided into at least 32 genotypes [17]. Interestingly, a single genotype of NoV GII.4 (genogroup II genotype 4) has been the predominant cause of major acute gastroenteritis epidemics in many countries since the mid-1990s [18], and the outbreak of GII.4 epidemics has increased in recent years [19]. Overall, the GII.4 genotype is estimated to be responsible for 60 to 80% of all NoV-associated outbreaks worldwide [20, 21].

As one of the largest developing countries, China has nearly one-fifth of the world's population and the second largest birth cohort in the world [22]. Currently, hospitalizations associated with NoV gastroenteritis are not uncommon in China [23–29]. Although scattered reports describing the epidemiology of NoVs in specific areas of China exist, the epidemiology and geographic distribution of NoV stains countrywide have not previously been described.

In the present study, all China-originating NoV sequences (partial and complete genome) during 1999–2011 (*n* = 983) were downloaded from GenBank. Genotyping and phylogenetic and recombination analyses of these sequences were performed in order to gain a better understanding of the distribution and genetic diversity of NoVs in China. The results provide a stronger basis for the evaluation and prevention of the spread and infection of NoVs in China.

## 2. Materials and Methods

### 2.1. Sequence Data Set

A total of 983 NoV sequences, derived from China, were retrieved from GenBank on June, 2012. They were isolated in between 1999 and 2011 (see Table S1, in Supplementary Material available online at http://dx.doi.org/10.1155/2014/196169, for detail). EditSeq (Lasergene software) was used to construct sequence file containing these 983 sequences and to edit the associated information for each sequence in a uniform format, for example, sequence name, sequence length, genotype, host, sample source, isolation time, and geographic area.

### 2.2. Genotyping Method and Phylogenetic Analysis

The genogroups, genotypes, and GII.4 variants of the NoV sequences were determined by the online-based typing-tool (http://www.rivm.nl/mpf/norovirus/typingtool) [30].

Nucleotide sequences were aligned using the ClustalW program. Phylogenetic analysis was performed with MEGA 5.1 package based on partial ORF1 (1029 nt) and ORF2 (811 nt) sequences. Phylogenetic trees were reconstructed using the Tamura-Nei model and maximum-likelihood method. Bootstrap was calculated with 1000 pseudoreplicate data sets. The distance scale represents the number of nucleotide substitution per position. A phylogenetic tree was first constructed using all of the GII.4 ORF1 sequences (*n* = 327) and the reference sequences retrieved from the NCBI database. Then, to simplify and clarify the complicated tree, a total of 41 representative sequences, which revealed, respectively, high similarity to other sequences grouped in the same cluster, were chosen to reconstruct the tree. Like the ORF1 tree, a total of 399 ORF2 sequences were analyzed initially, and then 108 representative sequences were used to reconstruct the phylogenetic tree.

### 2.3. Recombination Analysis

To detect recombination events, sequences were aligned using ClustalW and adjusted manually using SeqMan and BioEdit. The reference strains used were the same as those described above. The suspected recombinant strains were defined as recombinants if they were grouped into different genotypic clusters on the phylogenetic trees reconstructed using the 3′ end of ORF1 and the 5′ end of ORF2, respectively [31]. Phylogenetic analysis was performed as described above with the exception of 231 nt of ORF1 and 259 nt of ORF2 sequences.

SimPlot method was used to identify the breakpoint recombination site and to confirm the NoV recombinants [32]. The bootstrap values were plotted for a window of 300 bp, moving in increments of 10 bp along the alignment [32].

## 3. Results

### 3.1. Genetic Diversity of NoVs

**Figure 2:**
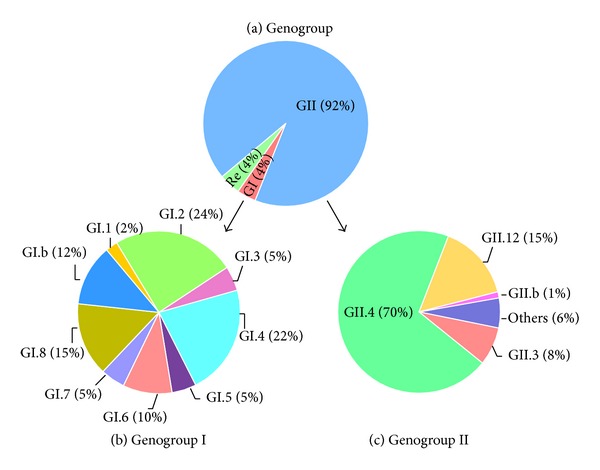
Diversity of NoV genotypes between 1999 and 2011 in China. (a) Genogroups of GI, GII and recombination (Re). (b) Genogroup I of GI.1, GI.2, GI.3, GI.4, GI.5, GI.6, GI.7, GI.8, and GI.b. (c) Genogroup II of GII.3, GII.4, GII.12, GII.b, and others (includes GII.2, GII.5, GII.6, GII.7, GII.8, GII.13, GII.14, GII.15, GII.16, GII.20, GII.21, and GII.a).

A total of 983 NoV nucleotide sequences from China were analyzed. Results indicated that more than 92.0% were genogroup II (GII), only 4.2% sequences were identified as genogroup I (GI), and the remaining 3.7% of sequences represented recombinant strains (Figure 2). Twenty-five genotypes of NoVs were identified: GI.1, GI.2, GI.3, GI.4, GI.5, GI.6, GI.7, GI.8, GI.b, GII.2, GII.3, GII.4, GII.5, GII.6, GII.7, GII.8, GII.12, GII.13, GII.14, GII.15, GII.16, GII.20, GII.21, GII.a, and GII.b. Nine genotypes belonged to GI, and 16 genotypes belonged to GII. In GII, the most predominant genotype was GII.4, which accounted for up to 70.0%, followed by GII.12 (15.1%), GII.3 (7.6%), and GII.b (1.2%) (Figure 2). GII.4 was also the most prevalent genotype among all 983 NoV sequences. In GI, the abundance of the 9 genotypes ranged from 24.4% (GI.2) to 2.4% (GI.1) (Figure 2).

**Figure 3:**
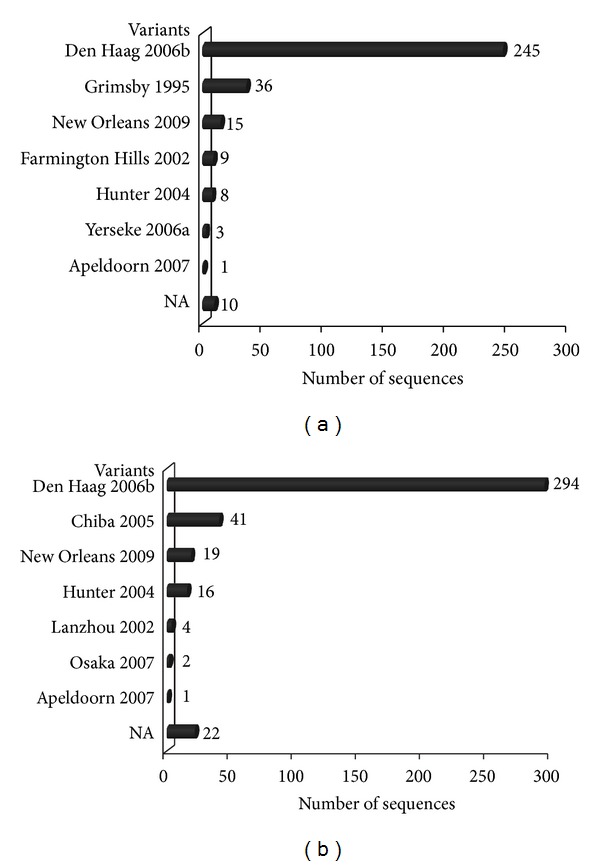
Distribution of NoV GII.4 variants based on ORF1 (a) and ORF2 (b). NA indicates the GII.4 sequences could not be assigned to any known variants.

A high diversity of the GII.4 variants was identified among all the NoV GII.4 sequences (*n* = 646) from 1999 to 2011. Based on ORF1 sequences, 327 sequences were grouped into 7 variant groups of 1996 (Grimsby 1995), 2002 (Farmington Hills 2002), 2004 (Hunter 2004), 2006a (Yerseke 2006a), 2006b (Den Haag 2006b), 2008 (Apeldoorn 2007), and 2010 variants (New Orleans 2009) (Figure 3(a)). The 2006b variant was the most predominant, accounting for nearly 75% of the GII.4 sequences (*n* = 327) (Figure 3(a)). Ten sequences could not be assigned to any known variants (Figure 3(a)). Based on ORF2 sequences, 399 sequences were also clustered into 7 variant groups, 2002CN (Lanzhou 2002), 2004 (Hunter 2004), 2005 (Chiba 2005), 2006b (Den Haag 2006b), 2007 (Osaka 2007), 2008 (Apeldoorn 2007), and 2010 (New Orleans 2009) (Figure 3(b)). The 2006b variants were also the most predominant, accounting for nearly 74% of the GII.4 sequences (*n* = 399) (Figure 3(b)). Twenty-two sequences could not be assigned to any known variants (Figure 3(b)). The 2003 and 2012 variants were found in neither the ORF2 nor ORF1 sequences.

**Figure 4:**
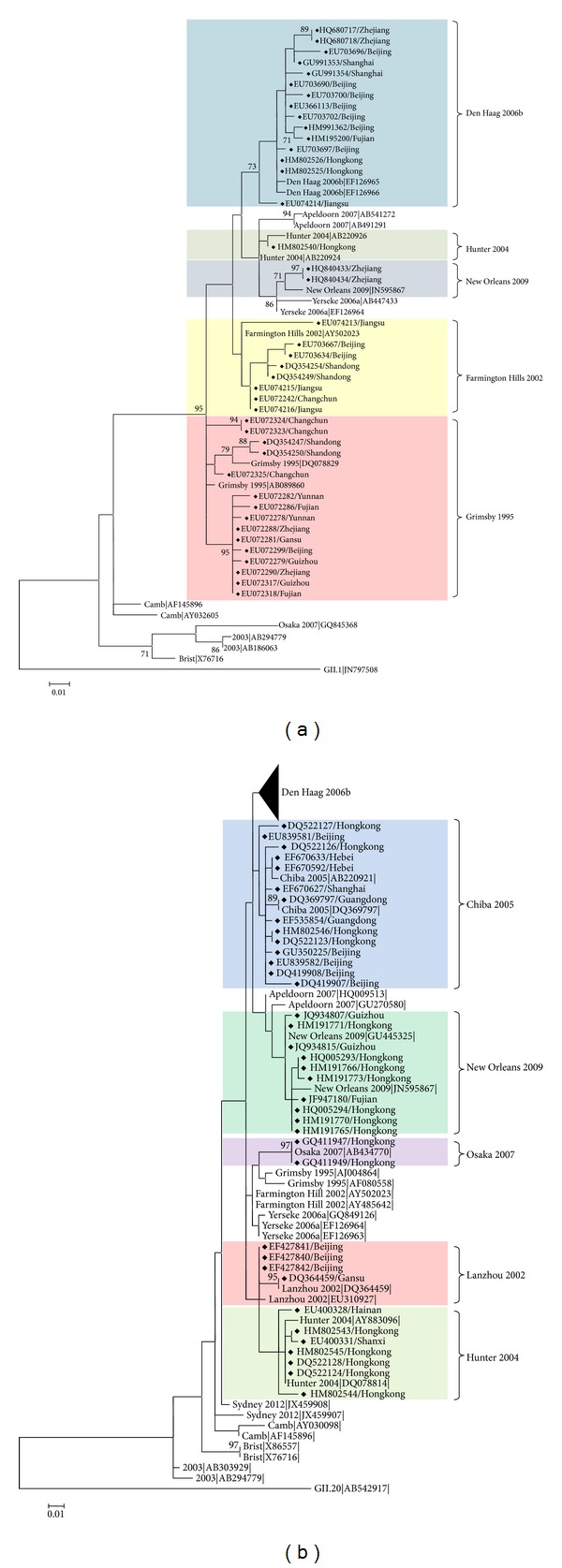
Phylogenetic analysis of GII.4 NoV sequences from China. Two rooted phylogenetic trees were reconstructed using partial ORF1 (1029 nt) (a) and ORF2 (811 nt) (b) nucleotide sequences. GII.1 (JN797508) and GII.20 (AB542917) were used as the outgroups in (a) and (b), respectively. The trees were generated using the maximum-likelihood method. Bootstrap values above 70%, estimated with 1000 pseudoreplicate data sets, are indicated at each node. The distance scale represents the number of nucleotide substitutions per position. The variant clusters are highlighted, and the NoV GII.4 sequences from China are indicated with a diamond. The reference sequences were retrieved from the NCBI database. The subtree of the Den Haag 2006b cluster in (b) includes 47 representative sequences.

Phylogenetic analysis of ORF1 showed that the NoV GII.4 sequences from China fell into 5 clusters, Den Haag 2006b, Hunter 2004, New Orleans 2009, Farmington Hills 2002, and Grimsby 1995 (Figure 4(a)). Of them, the Den Haag 2006b cluster consisted of a variety of strains oriented from distinct geographical locations, for example, Zhejiang, Beijing, Shanghai, Jiangsu, Fujian, and Hong Kong (Figure 4(a)). Similar results were also obtained from the phylogenetic tree of ORF2 (Figure 4(b)). The NoV GII.4 sequences from China formed 6 separate groups (Den Haag 2006b, Chiba 2005, New Orleans 2009, Osaka 2007, Lanzhou 2002, and Hunter 2004) (Figure 4(b)).

### 3.2. Geographic Distribution of NoV Genotypes

**Figure 5:**
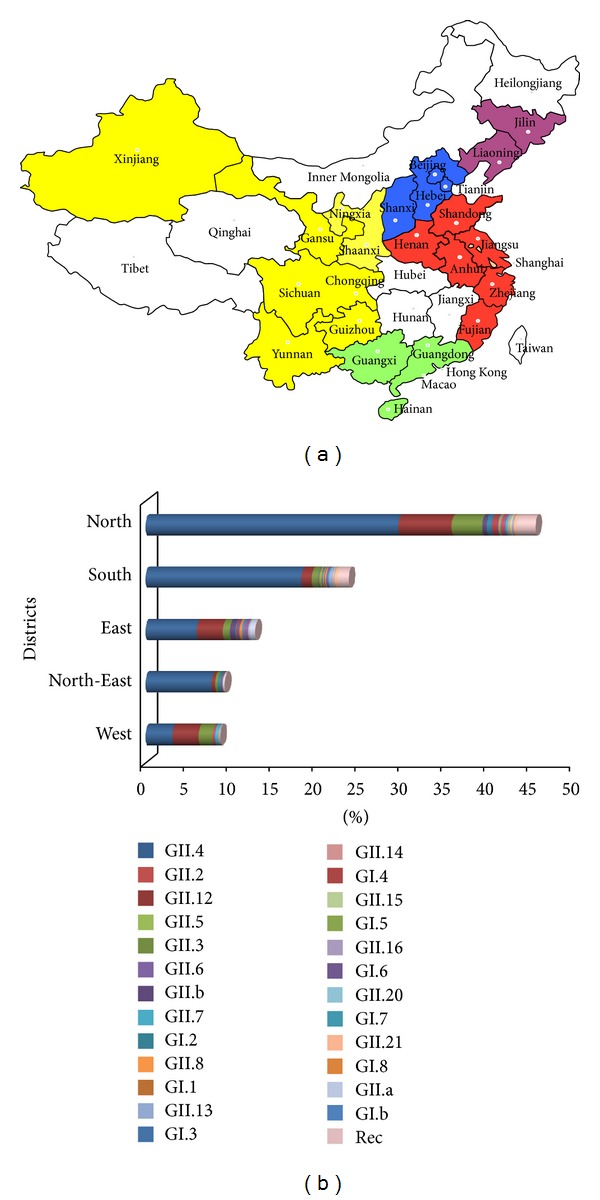
Geographical distribution of NoVs in China. (a) Sequence distribution map. The color-coding of each region is as follows: North China district (blue) includes Hebei, Shanxi, Beijing, and Tianjin; South China district (green) includes Guangdong, Guangxi, Hainan, and Hong Kong; East China district (red) includes Anhui, Shandong, Henan, Shanghai, Jiangsu, Zhejiang, and Fujian; North-East China district (purple) includes Jilin and Liaoning; West China district (yellow) includes Gansu, Xinjiang, Shanxi, Sichuan, Chongqing, Guizhou, and Yunnan. (b) Genotype distribution.

Geographically, NoV sequences were obtained from 24 provinces and municipalities in China, which can be further grouped into 5 main areas of East-China, West-China, South-China, North-China, and North-East China (Figure 5). The number of sequences from North-China was the highest (45.5%) among the 5 areas, followed by South-China (23.7%) and East-China (12.8%), dropping to 9.3% and 8.8% for North-East-China and West-China, respectively. Obviously, the majority of the NoV sequences were reported from the coastal regions (Figure 5(a)), such as Yellow Sea (Jilin, Liaoning, Beijing, Tianjin, and Shandong), East China Sea (Shanghai, Fujian, and Zhejiang), and South China Sea (Guangdong and Hong Kong). Interestingly, no NoV sequences were obtained from areas in central China, such as Hunan, Hubei, and Jiangxi provinces (Figure 5(a)).

Although the distribution of NoV genotypes was distinct among these 5 regions, in general, GII.4 was the most predominant genotype, followed by GII.12 and GII.3. The distribution of GII.4 was varied from 36.1% (West-China) to 83.5% (North-East China). Distribution of GII.12 ranged from 4.4% (North-East China) to 34.9% (West-China) and that of GII.3 ranged from 3.0% (South-China) to 18.6% (West-China) (Figure 5(b)).

### 3.3. Genetic Recombination of NoVs

**Figure 6:**
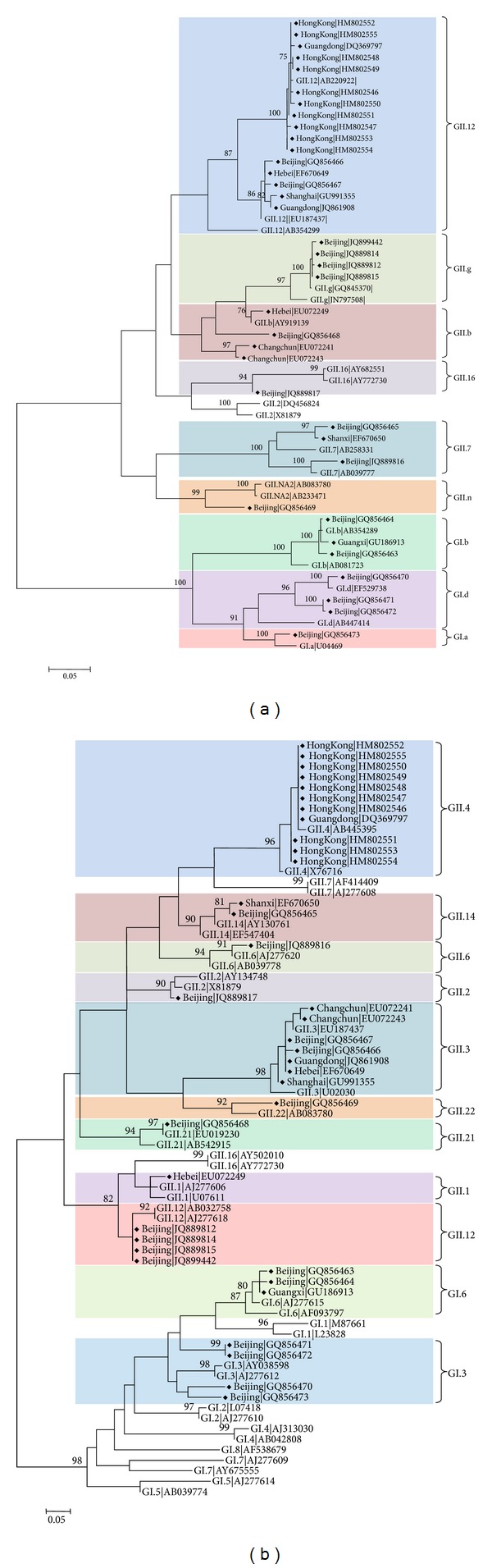
Phylogenetic recombination analyses of NoV sequences from China. Two unrooted phylogenetic trees were reconstructed using partial ORF1 (231 nt) (a) and ORF2 (259 nt) (b) nucleotide sequences. The trees were reconstructed using the maximum-likelihood method. Bootstrap values above 70%, estimated with 1000 pseudoreplicate data sets, are indicated at each node. The distance scale represents the number of nucleotide substitutions per position. The various genotypes are highlighted, and the recombination sequences from China are indicated with a diamond. The reference sequences were retrieved from the NCBI database.

Based on the NoV autotyping tool and phylogenetic analyses, 36 NoV sequences from China resulted from genetic recombination (Table 1 and Figure 6). Of these, 7 sequences were intergenotypic recombination of GI, and 29 sequences were intergenotypic recombination of GII. Inter-genogroup recombination was not detected (Table 1). The most predominant recombination type was GII.12/GII.4 (*n* = 11) and accounted for up to 30% of the 36 recombinant strains, of which 10 NoV sequences were collected from Hong Kong and 1 was from Guangdong (Table 1). One GII.4 2006a/2008 sequence from Hong Kong (NCBI accession number HQ005292) was identified as an intragenotype recombination type. It was clustered into 2 different variants of the 2006a and 2008 based on ORF1 and ORF2 phylogeny (data not shown).

In GI, 3 recombination types, GI.b/GI.6, GI.d/GI.3, and GI.a/GI.3, were observed, of which GI.6 and GI.3 were the dominant genotypes for the recombination that occurred on the ORF2 (Table 1 and Figure 6(b)). In GII, 10 recombination types were identified: GII.12/GII.3, GII.12/GII.4, GII.16/GII.2, GII.7/GII.14, GII.7/GII.6, GII.b/GII.21, GII.b/GII.3, GII.b/GII.1, GII.g/GII.12, and GII.n/GII.22, of which GII.12 was the most common genotype involved in recombination either on ORF1 or on ORF2 (Figure 6).

To further verify the recombination events, the SimPlot program was used, and the potential recombination sites were identified and further confirmed by boot scanning of the same genome sequences (data not shown).

As shown in Figure S1, all the recombinant NoVs were isolated in 4 main districts, for example, North China district (Beijing, Hebei, and Shanxi), South China district (Hong Kong, Guangdong, and Guangxi), North-East China district (Changchun), and East China district (Shanghai). Interestingly, nearly a half (*n* = 17) of the recombinants were obtained from Beijing, representing 10 out of the total of 13 recombinant genotypes detected in China (Table 1).

## 4. Discussion

The data presented in this study reveal a high genetic diversity of NoVs in China during the past 13 years. Most of the NoV genotypes (25 of 32) were found in China, and the predominant NoV (70.0%) was GII.4, which is the most common cause of outbreaks of acute gastroenteritis worldwide [20, 21]. Meanwhile, the prevalence of 2 other genotypes, for example, GII.12 and GII.3, was observed and also widely distributed all over the country.

### 4.1. Prevalence of GII.4 Variant

New subtypes or variants of GII.4 emerge every 1-2 years and usually become the dominant strains in the new seasons [18, 33]. Over the past 20 years, the GII.4 NoV has evolved a series of genetic variants, some of which persist and replace the previously circulating variants. The GII.4 2006b variant was the major player in the NoV outbreaks from 2006 to 2007 in Hong Kong and Japan [34, 35] and was the most predominant NoV variant in China until the end of 2011. A higher incidence of NoV outbreaks was observed worldwide in late 2012 [36], and the first molecular data from the outbreaks in Australia, France, New Zealand, and Japan available via NoroNet suggest that the outbreaks were related to emergence of a new variant of NoV GII.4, termed Sydney 2012 [36]. Amino acid changes were found in the main epitopes located at the P2 domain, which was consistent with the observations from prior epidemics [36]. The higher epidemiological fitness of the GII.4 strains has been attributed to a higher rate of evolution of the virus capsid proteins [37]. This may have led to an escape to existing herd immunity and might explain the observed increased outbreak activity [38].

### 4.2. Geographical Distribution of NoVs

NoVs were detected in most areas of China; however, both the number of NoV sequences and the diversity of NoV genotypes were distinct among different regions. Approximately 90% of NoV sequences were collected from the coastal regions, such as Liaoning, Beijing, Tianjin, Shandong, Jiangsu, Shanghai, Zhejiang, Fujian, Guangdong, and Hong Kong, which accounted for only one-fifth of the whole China. Why were most of the NoVs found in the coastal areas of China? One plausible speculation is linked to NoV-contaminated seafood. NoV outbreaks associated with oyster consumption have been well documented [39, 40]. Data from FAO Fisheries Statistics showed that the global production of oysters had reached more than 6 million tons from 2000 to 2010, and much of the production was pushed into the international market (http://www.fao.org/fishery/culturedspecies/Crassostrea_gigas/en). Accordingly, NoVs may spread worldwide during the international trade of contaminated oysters [41]. Cascading effects of NoV outbreaks usually spread to the entire community via the fecal-oral route or through the ingestion of contaminated food or water [42–44]. In addition, high population density and frequent communications of people from different regions and countries within the coastal regions of China may also attribute to the rapid spread of NoVs as well as increase the opportunity of NoV cross infection [40, 45]. However, it is worthwhile to note that the absence of NoV sequences in some regions, such as Inner Mongolia, Qinghai, Tibet, and Jiangxi, may result from the lack of surveillance studies.

Interestingly, most of the NoV sequences obtained from distinct geographical regions in China appeared to be closely related based on the phylogenetic analysis. For example, although the NoV GII.4 sequences were found in South, North, West, East, and North-East China, they were clustered into a single branch on the phylogenetic tree (Figure 4) instead of grouping into distinct lineages according to geographical locations. This suggests that NoVs spread and circulate rapidly in China rather than evolving in a region-specific manner. Due to the unbalanced development of the regional economy in China, numerous people from West China travel to the coastal areas, such as East China, South China, and North China, for work and back to their hometowns for the Spring Festival in the winter season every year. Accordingly, it is conceivable to speculate that the wide spread of NoVs results from this annual population movement, given that NoV-linked disease outbreaks often become prevalent during cold seasons, although the epidemiologic data of most NoV sequences are unavailable in China.

### 4.3. Diversity of NoV Recombinants

As recombination allows the virus to increase its genetic fitness, to evolve, and to spread in the host populations by escaping the host immune response [9], NoV recombinants of different genotypes and genogroups are widely distributed all over the world [46]. For example, a total of 28 outbreaks of NoV GI.b/GI.6 occurred in France (9 outbreaks), Hungary (1 outbreak), Sweden (3 outbreaks), Slovenia (2 outbreaks), United Kingdom (2 outbreaks), Denmark (5 outbreaks), Germany (4 outbreaks), and New Zealand (2 outbreaks) from 1995 to 2012 (http://www.rivm.nl/pubmpf/norovirus/database#/outbreaks/list). Additionally, in this study, 3 GI.b/GI.6 recombinants (GQ856463, GQ856464, and GU186913) were also identified both in Beijing and Guangxi, China.

Thus far, more than 20 NoV recombinant types have been identified [46, 47]. Of them, 13 recombinant types, including 3 GIs and 10 of GIIs, were identified in China based on phylogenetic analyses of the ORF1 and ORF2 of NoVs. It is notable that more than a half of the 983 sequences are less than 300 bp in length (Table S1), which may result in underestimation of the diversity of the recombination NoVs in China.

Our data also suggested a relatively high number (36 in 983 sequences) of recombinants were present in China-oriented NoV strains, and the number of involved GIIs (80%) was significantly higher than GIs (20%). In China, GII.12 is the most common genotype involved in the recombination both on ORF1 and ORF2, for example, GII.12/GII.3, GII.12/GII.4, and GII.g/GII.12. Coinfection of different NoV strains in a single host may provide the opportunity for recombination [48–50]. Previous studies confirmed the co-infection of different NoV strains in a single patient in several surveillance reports of NoV-associated outbreaks [48] and mixed infection of multiple strains in 1 outbreak [49, 50]. Therefore, high sequence conservation would increase the interaction between RNA molecules from different strains and recombination could occur as a result of homologous interaction [31, 47]. Although no direct evidence of recombination events resulting from a mixed infection of NoV strains exists in China, we found distinct genotypes in a single geographical location, such as Beijing, Shanghai, Guangdong, and Hong Kong. The circulation of a wide variety of different NoVs within a population in a given time period increases the potential for mixed infections which could lead to recombination.

Interestingly, some recombinant sequences revealed a geographical location-specific distribution pattern. For example, GII.12/GII.4 was only found in Guangdong and Hong Kong, and GII.12/GII.3 was found solely in Hebei and Beijing (Figure S1). Coincidently, all the geographical locations where the recombinants were detected also revealed a higher number of other NoV sequences, indicating that sequence recombination may occur. Since all the recombinant genotypes from China were found worldwide, more work needs to be done to provide further insights into their origin and spreading.

RNA homologous recombination is one of the crucial factors contributing to the genetic variation of NoVs [47, 51]. NoV recombination occurs frequently at the position of ORF1/ORF2 overlap [31]. Thus far, studies have demonstrated that the sequence of ORF1/ORF2 junction region in NoV is highly conserved [31, 47], and this region is considered as a negative-strand subgenomic RNA promoter site [47, 51]. The primary theory explaining RNA viruses' recombination is the copy-choice model [49, 51]. In this model, homologous recombination could occur during RNA replication with the involvement of viral RNA polymerase, which may switch the templates of 2 different strains at the highly conserved region of the genomes of the 2 strains. In the present study, the recombination breakpoint identified in all the sequences (both in GI and GII) was located at the ORF1/ORF2 junction region as well. Additionally, the recombination in NoV may confound the sequence identification and molecular epidemiologic studies. Classification of the NoVs should depend on both RdRp and capsid sequences rather than RdRp or capsid sequence [47].

In conclusion, the geographical distribution and genetic diversity of NoVs in China were studied in this work. Over the past 13 years, a high genetic diversity of NoVs was found, and the NoVs were widely but unevenly geographically distributed in most regions of China. These results may be helpful for the evaluation and implementation of appropriate measures for monitoring NoV infectious disease in China. Our findings also raise several questions on how the new variants spread so rapidly and what biological differences might have mediated the epidemic trend. Further work should focus on whether the disease caused by the new variants reveals more severe symptoms in patients or is more infectious. A global surveillance network may be needed to identify trends in molecular evolution of NoVs for prevention of future epidemics in China.

## Supplementary Material

Figure S1: Geographical distribution of NoV recombinant sequences from China. The color-coding of each region is as follows: The blue includes Hebei, Shanxi and Beijing; the green includes Guangdong and Guangxi; the red includes Shanghai; the purple includes Jilin.
Table S1: Information of 983 NoV sequences from China. The content includes viral sample isolation time, sequence submission time, geographical origin, host, reference and sequence genotyping results.

## Figures and Tables

**Table 1:** Recombinants of NoVs from China.

ORF1 genotype	ORF2 genotype	Accession number	Sequence length (nt)	Geographic location	ORF1 genotype	ORF2 genotype	Accession number	Sequence length (nt)	Geographic location
I.a	I.3	GQ856473	3173	Beijing	I.b	I.6	GU186913	595	Guangxi
I.b	I.6	GQ856463	3109	Beijing	II.b	II.3	EU072241	3227	Jilin
I.b	I.6	GQ856464	3109	Beijing	II.b	II.3	EU072243	3230	Jilin
I.d	I.3	GQ856470	3084	Beijing	II.b	II.1	EU072249	3188	Hebei
I.d	I.3	GQ856471	3132	Beijing	II.12	II.3	EF670649	3230	Hebei
I.d	I.3	GQ856472	3132	Beijing	II.12	II.4	HM802546	3988	Hong Kong
II.b	II.21	GQ856468	3174	Beijing	II.12	II.4	HM802547	3988	Hong Kong
II.g	II.12	JQ889812	1009	Beijing	II.12	II.4	HM802548	3988	Hong Kong
II.g	II.12	JQ889814	1009	Beijing	II.12	II.4	HM802549	3988	Hong Kong
II.g	II.12	JQ889815	1009	Beijing	II.12	II.4	HM802550	3988	Hong Kong
II.g	II.12	JQ899442	1009	Beijing	II.12	II.4	HM802551	3988	Hong Kong
II.n	II.22	GQ856469	3061	Beijing	II.12	II.4	HM802552	3988	Hong Kong
II.7	II.6	JQ889816	1009	Beijing	II.12	II.4	HM802553	3988	Hong Kong
II.7	II.14	GQ856465	3237	Beijing	II.12	II.4	HM802554	3988	Hong Kong
II.12	II.3	GQ856466	3230	Beijing	II.12	II.4	HM802555	3988	Hong Kong
II.12	II.3	GQ856467	3230	Beijing	II.12	II.3	JQ861908	583	Guangdong
II.16	II.2	JQ889817	1009	Beijing	II.12	II.4	DQ369797	7558	Guangdong
II.7	II.14	EF670650	7359	Shanxi	II.12	II.3	GU991355	7544	Shanghai
