# Supplementary material for: Genetic Diversity and Distribution of Human Norovirus in China (1999–2011)
Source: Biomed Res Int. 2014 Jan 21;2014:196169. doi: 10.1155/2014/196169 (PMC3918700; doi:10.1155/2014/196169)
Supplement: Supplementary file 1 — Figure S1: Geographical distribution of NoV recombinant sequences from China. The color-coding of each region is as follows: The blue includes Hebei, Shanxi and Beijing; the green includes Guangdong and Guangxi; the red includes Shanghai; the purple includes Jilin. Table S1: Information of 983 NoV sequences from China. The content includes viral sample isolation time, sequence submission time, geographical origin, host, reference and sequence genotyping results. [file 196169.f1.pdf]

# Supplementary materials

For submission to *BioMed Research International*

**Revised version**

## Genetic diversity and distribution of human norovirus in China (1999-2011)

Yongxin Yu[1,2], Shuling Yan[2,3], Bailin Li[1,2], Yingjie Pan[1,2], Yongjie Wang[1,2]*

[1] Shanghai Engineering Research Center of Aquatic-Product Processing & Preservation, China; [2] College of Food Science and Technology, Shanghai Ocean University, Shanghai, China; [3] Institute of Biochemistry and Molecular Cell Biology, University of Goettingen, Goettingen, Germany

**Running title: Diversity of norovirus**

Number of figure: 1; number of table: 1

* Corresponding author:

Tel.: +86 21 61900505

Email: yjwang@shou.edu.cn (Y. Wang)

**Figure S1** Geographical distribution of NoV recombinant sequences from China

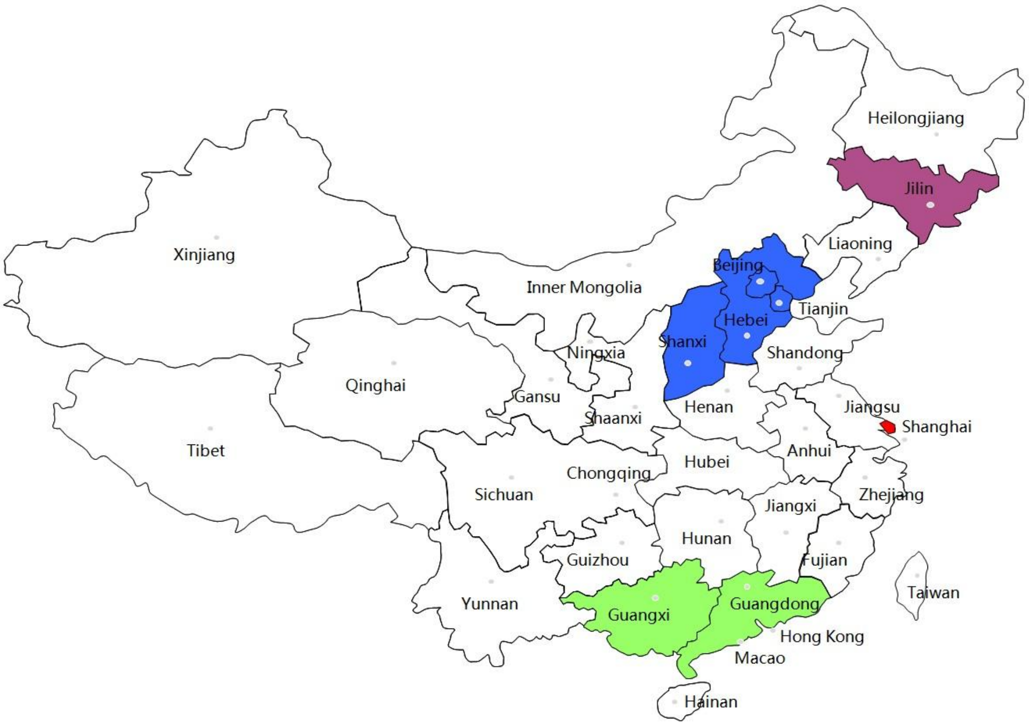

**Table S1** Information of 983 NoV sequences from China

| No. | Province | Accession No. | Length (bp) | ORF1 | ORF2 | Host | Sample | Submission year | Isolation year | Reference |
|---|---|---|---|---|---|---|---|---|---|---|
| 1 | Anhui | EU072215 | 274 | II.3 | | human | stool | 2007 | 1999~2005 | unpublished |
| 2 | Anhui | EU072227 | 274 | II.3 | | human | stool | 2007 | 1999~2005 | unpublished |
| 3 | Anhui | EU072228 | 274 | I.2 | | human | stool | 2007 | 1999~2005 | unpublished |
| 4 | Anhui | EU072229 | 274 | II.12 | | human | stool | 2007 | 1999~2005 | unpublished |
| 5 | Anhui | EU072231 | 274 | II.12 | | human | stool | 2007 | 1999~2005 | unpublished |
| 6 | Anhui | EU072232 | 274 | II.12 | | human | stool | 2007 | 1999~2005 | unpublished |
| 7 | Anhui | EU072233 | 274 | II.12 | | human | stool | 2007 | 1999~2005 | unpublished |
| 8 | Anhui | EU072234 | 274 | II.12 | | human | stool | 2007 | 1999~2005 | unpublished |
| 9 | Anhui | EU072235 | 2993 | II.20 | II.20 | human | stool | 2007 | 1999~2005 | unpublished |
| 10 | Anhui | EU400360 | 281 | | II.4 | human | stool | 2008 | 2006 | 1 |
| 11 | Anhui | EU400361 | 281 | | II.4 | human | stool | 2008 | 2006 | 1 |
| 12 | Anhui | EU400362 | 281 | | II.4 | human | stool | 2008 | 2006 | 1 |
| 13 | Anhui | EU400363 | 281 | | II.4 | human | stool | 2008 | 2006 | 1 |
| 14 | Anhui | EU400364 | 281 | | II.4 | human | stool | 2008 | 2006 | 1 |
| 15 | Anhui | EU400381 | 297 | | I.4 | human | stool | 2008 | 2006 | 1 |
| 16 | Beijing | DQ419907 | 1620 | | II.4 | human | stool | 2006 | 2004 | unpublished |
| 17 | Beijing | DQ419908 | 1623 | | II.4 | human | stool | 2006 | 2004 | unpublished |
| 18 | Beijing | DQ419909 | 1647 | | II.3 | human | stool | 2006 | 2004 | unpublished |
| 19 | Beijing | EF427840 | 285 | | II.4 | human | stool | 2007 | 2002 | unpublished |
| 20 | Beijing | EF427841 | 261 | | II.4 | human | stool | 2007 | 2002 | unpublished |
| 21 | Beijing | EF427842 | 285 | | II.4 | human | stool | 2007 | 2002 | unpublished |
| 22 | Beijing | EF427843 | 297 | | I.4 | human | stool | 2007 | 2002 | unpublished |
| 23 | Beijing | EU072219 | 274 | II.12 | | human | unclear | 2007 | 1999~2005 | unpublished |
| 24 | Beijing | EU072220 | 274 | II.6 | | human | unclear | 2007 | 1999~2005 | unpublished |
| 25 | Beijing | EU072299 | 274 | II.4 | | human | unclear | 2007 | 1999~2005 | unpublished |
| 26 | Beijing | EU072301 | 274 | II.a | | human | unclear | 2007 | 1999~2005 | unpublished |
| 27 | Beijing | EU072302 | 274 | II.3 | | human | unclear | 2007 | 1999~2005 | unpublished |
| 28 | Beijing | EU072303 | 274 | II.3 | | human | unclear | 2007 | 1999~2005 | unpublished |
| 29 | Beijing | EU072319 | 274 | II.8 | | human | unclear | 2007 | 1999~2005 | unpublished |
| 30 | Beijing | EU072341 | 274 | II.7 | | human | unclear | 2007 | 1999~2005 | unpublished |
| 31 | Beijing | EU366113 | 3282 | II.4 | II.4 | human | unclear | 2007 | 2006 | unpublished |
| 32 | Beijing | EU482093 | 674 | | II.4 | human | unclear | 2008 | 2007 | unpublished |
| 33 | Beijing | EU482094 | 610 | | II.4 | human | unclear | 2008 | 2007 | unpublished |
| 34 | Beijing | EU482095 | 624 | | II.4 | human | unclear | 2008 | 2007 | unpublished |
| 35 | Beijing | EU703630 | 319 | II.12 | | human | stool | 2008 | 2004 | 5 |
| 36 | Beijing | EU703631 | 319 | II.12 | | human | stool | 2008 | 2004 | 5 |
| 37 | Beijing | EU703632 | 319 | II.12 | | human | stool | 2008 | 2004 | 5 |
| 38 | Beijing | EU703633 | 319 | II.12 | | human | stool | 2008 | 2004 | 5 |
| 39 | Beijing | EU703634 | 319 | II.4 | | human | stool | 2008 | 2004 | 5 |

| | | | | | | | | | | |
|---|---|---|---|---|---|---|---|---|---|---|
| 40 | Beijing | EU703635 | 319 | II.b | | human | stool | 2008 | 2004 | 5 |
| 41 | Beijing | EU703636 | 319 | II.12 | | human | stool | 2008 | 2004 | 5 |
| 42 | Beijing | EU703637 | 319 | II.12 | | human | stool | 2008 | 2004 | 5 |
| 43 | Beijing | EU703638 | 319 | II.12 | | human | stool | 2008 | 2004 | 5 |
| 44 | Beijing | EU703639 | 319 | II.12 | | human | stool | 2008 | 2004 | 5 |
| 45 | Beijing | EU703640 | 319 | II.3 | | human | stool | 2008 | 2004 | 5 |
| 46 | Beijing | EU703641 | 319 | II.12 | | human | stool | 2008 | 2004 | 5 |
| 47 | Beijing | EU703642 | 319 | II.12 | | human | stool | 2008 | 2004 | 5 |
| 48 | Beijing | EU703643 | 319 | II.12 | | human | stool | 2008 | 2004 | 5 |
| 49 | Beijing | EU703644 | 319 | II.b | | human | stool | 2008 | 2004 | 5 |
| 50 | Beijing | EU703645 | 319 | II.12 | | human | stool | 2008 | 2005 | 5 |
| 51 | Beijing | EU703646 | 319 | II.12 | | human | stool | 2008 | 2005 | 5 |
| 52 | Beijing | EU703647 | 319 | II.12 | | human | stool | 2008 | 2005 | 5 |
| 53 | Beijing | EU703648 | 319 | II.12 | | human | stool | 2008 | 2005 | 5 |
| 54 | Beijing | EU703649 | 319 | II.12 | | human | stool | 2008 | 2005 | 5 |
| 55 | Beijing | EU703650 | 319 | II.12 | | human | stool | 2008 | 2005 | 5 |
| 56 | Beijing | EU703651 | 319 | II.12 | | human | stool | 2008 | 2005 | 5 |
| 57 | Beijing | EU703652 | 319 | II.4 | | human | stool | 2008 | 2005 | 5 |
| 58 | Beijing | EU703653 | 319 | II.4 | | human | stool | 2008 | 2005 | 5 |
| 59 | Beijing | EU703654 | 319 | II.12 | | human | stool | 2008 | 2005 | 5 |
| 60 | Beijing | EU703655 | 319 | II.12 | | human | stool | 2008 | 2005 | 5 |
| 61 | Beijing | EU703656 | 319 | II.12 | | human | stool | 2008 | 2005 | 5 |
| 62 | Beijing | EU703657 | 319 | II.12 | | human | stool | 2008 | 2005 | 5 |
| 63 | Beijing | EU703658 | 319 | II.12 | | human | stool | 2008 | 2005 | 5 |
| 64 | Beijing | EU703659 | 319 | II.12 | | human | stool | 2008 | 2005 | 5 |
| 65 | Beijing | EU703660 | 319 | II.12 | | human | stool | 2008 | 2005 | 5 |
| 66 | Beijing | EU703661 | 319 | II.2 | | human | stool | 2008 | 2005 | 5 |
| 67 | Beijing | EU703662 | 319 | II.4 | | human | stool | 2008 | 2005 | 5 |
| 68 | Beijing | EU703663 | 319 | II.12 | | human | stool | 2008 | 2005 | 5 |
| 69 | Beijing | EU703664 | 319 | II.12 | | human | stool | 2008 | 2005 | 5 |
| 70 | Beijing | EU703665 | 319 | II.4 | | human | stool | 2008 | 2005 | 5 |
| 71 | Beijing | EU703666 | 319 | II.12 | | human | stool | 2008 | 2005 | 5 |
| 72 | Beijing | EU703667 | 319 | II.4 | | human | stool | 2008 | 2005 | 5 |
| 73 | Beijing | EU703668 | 319 | II.12 | | human | stool | 2008 | 2005 | 5 |
| 74 | Beijing | EU703669 | 319 | II.12 | | human | stool | 2008 | 2005 | 5 |
| 75 | Beijing | EU703670 | 319 | II.12 | | human | stool | 2008 | 2005 | 5 |
| 76 | Beijing | EU703671 | 319 | II.12 | | human | stool | 2008 | 2005 | 5 |
| 77 | Beijing | EU703672 | 319 | II.12 | | human | stool | 2008 | 2005 | 5 |
| 78 | Beijing | EU703673 | 319 | II.4 | | human | stool | 2008 | 2005 | 5 |
| 79 | Beijing | EU703674 | 319 | II.12 | | human | stool | 2008 | 2005 | 5 |
| 80 | Beijing | EU703675 | 319 | II.12 | | human | stool | 2008 | 2005 | 5 |
| 81 | Beijing | EU703676 | 319 | II.12 | | human | stool | 2008 | 2005 | 5 |
| 82 | Beijing | EU703677 | 319 | II.12 | | human | stool | 2008 | 2005 | 5 |

| | | | | | | | | | | |
|---|---|---|---|---|---|---|---|---|---|---|
| 83 | Beijing | EU703678 | 319 | II.12 | | human | stool | 2008 | 2005 | 5 |
| 84 | Beijing | EU703679 | 319 | II.12 | | human | stool | 2008 | 2005 | 5 |
| 85 | Beijing | EU703680 | 319 | II.12 | | human | stool | 2008 | 2005 | 5 |
| 86 | Beijing | EU703681 | 319 | II.12 | | human | stool | 2008 | 2005 | 5 |
| 87 | Beijing | EU703682 | 319 | II.12 | | human | stool | 2008 | 2005 | 5 |
| 88 | Beijing | EU703683 | 319 | II.12 | | human | stool | 2008 | 2005 | 5 |
| 89 | Beijing | EU703684 | 319 | II.12 | | human | stool | 2008 | 2005 | 5 |
| 90 | Beijing | EU703685 | 319 | II.12 | | human | stool | 2008 | 2005 | 5 |
| 91 | Beijing | EU703686 | 319 | II.12 | | human | stool | 2008 | 2005 | 5 |
| 92 | Beijing | EU703687 | 319 | II.12 | | human | stool | 2008 | 2005 | 5 |
| 93 | Beijing | EU703688 | 319 | II.12 | | human | stool | 2008 | 2005 | 5 |
| 94 | Beijing | EU703689 | 319 | II.12 | | human | stool | 2008 | 2005 | 5 |
| 95 | Beijing | EU703690 | 319 | II.4 | | human | stool | 2008 | 2006 | 5 |
| 96 | Beijing | EU703691 | 319 | II.12 | | human | stool | 2008 | 2006 | 5 |
| 97 | Beijing | EU703692 | 319 | II.4 | | human | stool | 2008 | 2006 | 5 |
| 98 | Beijing | EU703693 | 319 | II.12 | | human | stool | 2008 | 2006 | 5 |
| 99 | Beijing | EU703694 | 319 | II.12 | | human | stool | 2008 | 2006 | 5 |
| 100 | Beijing | EU703695 | 319 | II.12 | | human | stool | 2008 | 2006 | 5 |
| 101 | Beijing | EU703696 | 319 | II.4 | | human | stool | 2008 | 2007 | 5 |
| 102 | Beijing | EU703697 | 319 | II.4 | | human | stool | 2008 | 2007 | 5 |
| 103 | Beijing | EU703698 | 319 | II.4 | | human | stool | 2008 | 2007 | 5 |
| 104 | Beijing | EU703699 | 319 | II.4 | | human | stool | 2008 | 2007 | 5 |
| 105 | Beijing | EU703700 | 319 | II.4 | | human | stool | 2008 | 2007 | 5 |
| 106 | Beijing | EU703701 | 319 | II.4 | | human | stool | 2008 | 2007 | 5 |
| 107 | Beijing | EU703702 | 319 | II.4 | | human | stool | 2008 | 2007 | 5 |
| 108 | Beijing | EU703703 | 319 | II.4 | | human | stool | 2008 | 2007 | 5 |
| 109 | Beijing | EU703704 | 319 | II.4 | | human | stool | 2008 | 2007 | 5 |
| 110 | Beijing | EU703705 | 319 | II.4 | | human | stool | 2008 | 2007 | 5 |
| 111 | Beijing | EU703706 | 319 | II.4 | | human | stool | 2008 | 2007 | 5 |
| 112 | Beijing | EU703707 | 319 | II.4 | | human | stool | 2008 | 2007 | 5 |
| 113 | Beijing | EU703708 | 319 | II.4 | | human | stool | 2008 | 2007 | 5 |
| 114 | Beijing | EU703709 | 319 | II.4 | | human | stool | 2008 | 2007 | 5 |
| 115 | Beijing | EU703710 | 319 | II.2 | | human | stool | 2008 | 2007 | 5 |
| 116 | Beijing | EU703711 | 319 | II.4 | | human | stool | 2008 | 2006 | 5 |
| 117 | Beijing | EU703712 | 319 | II.4 | | human | stool | 2008 | 2006 | 5 |
| 118 | Beijing | EU703713 | 319 | II.12 | | human | stool | 2008 | 2006 | 5 |
| 119 | Beijing | EU703714 | 319 | II.4 | | human | stool | 2008 | 2007 | 5 |
| 120 | Beijing | EU703715 | 319 | II.4 | | human | stool | 2008 | 2007 | 5 |
| 121 | Beijing | EU703716 | 319 | II.4 | | human | stool | 2008 | 2007 | 5 |
| 122 | Beijing | EU703717 | 319 | II.4 | | human | stool | 2008 | 2007 | 5 |
| 123 | Beijing | EU703718 | 319 | II.4 | | human | stool | 2008 | 2007 | 5 |
| 124 | Beijing | EU703719 | 319 | II.4 | | human | stool | 2008 | 2007 | 5 |
| 125 | Beijing | EU703720 | 319 | II.4 | | human | stool | 2008 | 2007 | 5 |

| | | | | | | | | | | |
|---|---|---|---|---|---|---|---|---|---|---|
| 126 | Beijing | EU703721 | 319 | II.4 | | human | stool | 2008 | 2007 | 5 |
| 127 | Beijing | EU703722 | 319 | II.4 | | human | stool | 2008 | 2007 | 5 |
| 128 | Beijing | EU703723 | 319 | II.4 | | human | stool | 2008 | 2007 | 5 |
| 129 | Beijing | EU703724 | 319 | II.4 | | human | stool | 2008 | 2007 | 5 |
| 130 | Beijing | EU703725 | 319 | II.4 | | human | stool | 2008 | 2007 | 5 |
| 131 | Beijing | EU703726 | 319 | II.4 | | human | stool | 2008 | 2007 | 5 |
| 132 | Beijing | EU703727 | 319 | II.4 | | human | stool | 2008 | 2007 | 5 |
| 133 | Beijing | EU703728 | 319 | II.4 | | human | stool | 2008 | 2007 | 5 |
| 134 | Beijing | EU703729 | 319 | II.4 | | human | stool | 2008 | 2007 | 5 |
| 135 | Beijing | EU703730 | 344 | | II.3 | human | stool | 2008 | 2005 | 5 |
| 136 | Beijing | EU703731 | 344 | | II.3 | human | stool | 2008 | 2005 | 5 |
| 137 | Beijing | EU703732 | 344 | | II.4 | human | stool | 2008 | 2005 | 5 |
| 138 | Beijing | EU703733 | 344 | | II.3 | human | stool | 2008 | 2005 | 5 |
| 139 | Beijing | EU703734 | 344 | | II.3 | human | stool | 2008 | 2005 | 5 |
| 140 | Beijing | EU703735 | 344 | | II.3 | human | stool | 2008 | 2005 | 5 |
| 141 | Beijing | EU703736 | 344 | | II.4 | human | stool | 2008 | 2005 | 5 |
| 142 | Beijing | EU703737 | 344 | | II.3 | human | stool | 2008 | 2005 | 5 |
| 143 | Beijing | EU703738 | 344 | | II.3 | human | stool | 2008 | 2005 | 5 |
| 144 | Beijing | EU703739 | 344 | | II.3 | human | stool | 2008 | 2005 | 5 |
| 145 | Beijing | EU703740 | 344 | | II.3 | human | stool | 2008 | 2005 | 5 |
| 146 | Beijing | EU703741 | 344 | | II.3 | human | stool | 2008 | 2005 | 5 |
| 147 | Beijing | EU703742 | 344 | | II.4 | human | stool | 2008 | 2005 | 5 |
| 148 | Beijing | EU703743 | 344 | | II.3 | human | stool | 2008 | 2005 | 5 |
| 149 | Beijing | EU703744 | 344 | | II.3 | human | stool | 2008 | 2006 | 5 |
| 150 | Beijing | EU703745 | 344 | | II.4 | human | stool | 2008 | 2007 | 5 |
| 151 | Beijing | EU703746 | 344 | | II.4 | human | stool | 2008 | 2007 | 5 |
| 152 | Beijing | EU703747 | 344 | | II.4 | human | stool | 2008 | 2007 | 5 |
| 153 | Beijing | EU703748 | 344 | | II.4 | human | stool | 2008 | 2007 | 5 |
| 154 | Beijing | EU703749 | 344 | | II.4 | human | stool | 2008 | 2007 | 5 |
| 155 | Beijing | EU703750 | 344 | | II.4 | human | stool | 2008 | 2007 | 5 |
| 156 | Beijing | EU703751 | 344 | | II.4 | human | stool | 2008 | 2007 | 5 |
| 157 | Beijing | EU703752 | 344 | | II.4 | human | stool | 2008 | 2007 | 5 |
| 158 | Beijing | EU703753 | 344 | | II.3 | human | stool | 2008 | 2007 | 5 |
| 159 | Beijing | EU703754 | 344 | | II.4 | human | stool | 2008 | 2007 | 5 |
| 160 | Beijing | EU703755 | 344 | | II.4 | human | stool | 2008 | 2007 | 5 |
| 161 | Beijing | EU703756 | 344 | | II.4 | human | stool | 2008 | 2007 | 5 |
| 162 | Beijing | EU703757 | 344 | | II.4 | human | stool | 2008 | 2007 | 5 |
| 163 | Beijing | EU703758 | 344 | | II.4 | human | stool | 2008 | 2007 | 5 |
| 164 | Beijing | EU839581 | 1623 | | II.4 | human | stool | 2008 | 2004 | 5 |
| 165 | Beijing | EU839582 | 1623 | | II.4 | human | stool | 2008 | 2004 | 5 |
| 166 | Beijing | EU839583 | 1623 | | II.4 | human | stool | 2008 | 2004 | 5 |
| 167 | Beijing | EU839584 | 1623 | | II.4 | human | stool | 2008 | 2005 | 5 |
| 168 | Beijing | EU839585 | 1623 | | II.4 | human | stool | 2008 | 2005 | 5 |

| | | | | | | | | | | |
|---|---|---|---|---|---|---|---|---|---|---|
| 169 | Beijing | EU839586 | 1623 | | II.4 | human | stool | 2008 | 2005 | 5 |
| 170 | Beijing | EU839587 | 1623 | | II.4 | human | stool | 2008 | 2005 | 5 |
| 171 | Beijing | EU839588 | 1623 | | II.4 | human | stool | 2008 | 2006 | 5 |
| 172 | Beijing | EU839589 | 1623 | | II.4 | human | stool | 2008 | 2006 | 5 |
| 173 | Beijing | EU839590 | 1623 | | II.4 | human | stool | 2008 | 2007 | 5 |
| 174 | Beijing | EU839591 | 1623 | | II.4 | human | stool | 2008 | 2007 | 5 |
| 175 | Beijing | EU839592 | 1623 | | II.4 | human | stool | 2008 | 2007 | 5 |
| 176 | Beijing | EU839593 | 1623 | | II.4 | human | stool | 2008 | 2007 | 5 |
| 177 | Beijing | EU839594 | 1623 | | II.4 | human | stool | 2008 | 2007 | 5 |
| 178 | Beijing | EU839595 | 1623 | | II.4 | human | stool | 2008 | 2007 | 5 |
| 179 | Beijing | EU850823 | 1647 | | II.3 | human | stool | 2008 | 2005 | 5 |
| 180 | Beijing | EU850824 | 1647 | | II.3 | human | stool | 2008 | 2005 | 5 |
| 181 | Beijing | EU850825 | 1647 | | II.3 | human | stool | 2008 | 2005 | 5 |
| 182 | Beijing | EU850826 | 1647 | | II.3 | human | stool | 2008 | 2005 | 5 |
| 183 | Beijing | EU850827 | 1647 | | II.3 | human | stool | 2008 | 2005 | 5 |
| 184 | Beijing | GQ380642 | 280 | | I.8 | human | stool | 2009 | 2007 | 8 |
| 185 | Beijing | GQ380643 | 246 | | I.4 | human | stool | 2009 | 2007 | 8 |
| 186 | Beijing | GQ380644 | 252 | | I.4 | human | stool | 2009 | 2007 | 8 |
| 187 | Beijing | GQ380645 | 358 | | II.4 | human | stool | 2009 | 2007 | 8 |
| 188 | Beijing | GQ380646 | 358 | | II.4 | human | stool | 2009 | 2007 | 8 |
| 189 | Beijing | GQ380647 | 361 | | II.4 | human | stool | 2009 | 2007 | 8 |
| 190 | Beijing | GQ380648 | 358 | | II.4 | human | stool | 2009 | 2007 | 8 |
| 191 | Beijing | GQ380649 | 357 | | II.4 | human | stool | 2009 | 2007 | 8 |
| 192 | Beijing | GQ380650 | 360 | | II.4 | human | stool | 2009 | 2007 | 8 |
| 193 | Beijing | GQ380651 | 357 | | II.21 | human | stool | 2009 | 2007 | 8 |
| 194 | Beijing | GQ380652 | 363 | | II.3 | human | stool | 2009 | 2008 | 8 |
| 195 | Beijing | GQ380653 | 361 | | II.4 | human | stool | 2009 | 2008 | 8 |
| 196 | Beijing | GQ380654 | 360 | | II.4 | human | stool | 2009 | 2008 | 8 |
| 197 | Beijing | GQ380655 | 359 | | II.4 | human | stool | 2009 | 2008 | 8 |
| 198 | Beijing | GQ856371 | 247 | II.4 | | human | stool | 2009 | 2007 | 11 |
| 199 | Beijing | GQ856372 | 247 | II.4 | | human | stool | 2009 | 2007 | 11 |
| 200 | Beijing | GQ856373 | 247 | II.4 | | human | stool | 2009 | 2007 | 11 |
| 201 | Beijing | GQ856374 | 247 | II.4 | | human | stool | 2009 | 2007 | 11 |
| 202 | Beijing | GQ856375 | 247 | II.4 | | human | stool | 2009 | 2007 | 11 |
| 203 | Beijing | GQ856376 | 247 | II.4 | | human | stool | 2009 | 2007 | 11 |
| 204 | Beijing | GQ856377 | 247 | II.4 | | human | stool | 2009 | 2007 | 11 |
| 205 | Beijing | GQ856378 | 247 | II.4 | | human | stool | 2009 | 2007 | 11 |
| 206 | Beijing | GQ856379 | 247 | II.4 | | human | stool | 2009 | 2007 | 11 |
| 207 | Beijing | GQ856380 | 247 | II.4 | | human | stool | 2009 | 2007 | 11 |
| 208 | Beijing | GQ856381 | 247 | II.4 | | human | stool | 2009 | 2007 | 11 |
| 209 | Beijing | GQ856382 | 212 | II.4 | | human | stool | 2009 | 2007 | 11 |
| 210 | Beijing | GQ856383 | 247 | II.4 | | human | stool | 2009 | 2007 | 11 |
| 211 | Beijing | GQ856384 | 212 | II.4 | | human | stool | 2009 | 2007 | 11 |

| | | | | | | | | | | |
|---|---|---|---|---|---|---|---|---|---|---|
| 212 | Beijing | GQ856385 | 247 | II.4 | | human | stool | 2009 | 2007 | 11 |
| 213 | Beijing | GQ856386 | 247 | II.4 | | human | stool | 2009 | 2007 | 11 |
| 214 | Beijing | GQ856387 | 247 | II.4 | | human | stool | 2009 | 2007 | 11 |
| 215 | Beijing | GQ856388 | 247 | II.4 | | human | stool | 2009 | 2007 | 11 |
| 216 | Beijing | GQ856389 | 247 | II.4 | | human | stool | 2009 | 2007 | 11 |
| 217 | Beijing | GQ856390 | 247 | II.4 | | human | stool | 2009 | 2007 | 11 |
| 218 | Beijing | GQ856391 | 247 | II.4 | | human | stool | 2009 | 2007 | 11 |
| 219 | Beijing | GQ856392 | 247 | II.4 | | human | stool | 2009 | 2007 | 11 |
| 220 | Beijing | GQ856393 | 247 | II.4 | | human | stool | 2009 | 2007 | 11 |
| 221 | Beijing | GQ856394 | 247 | II.4 | | human | stool | 2009 | 2007 | 11 |
| 222 | Beijing | GQ856395 | 247 | II.4 | | human | stool | 2009 | 2007 | 11 |
| 223 | Beijing | GQ856396 | 247 | II.4 | | human | stool | 2009 | 2007 | 11 |
| 224 | Beijing | GQ856397 | 247 | II.4 | | human | stool | 2009 | 2007 | 11 |
| 225 | Beijing | GQ856398 | 212 | II.4 | | human | stool | 2009 | 2007 | 11 |
| 226 | Beijing | GQ856399 | 212 | II.4 | | human | stool | 2009 | 2007 | 11 |
| 227 | Beijing | GQ856400 | 212 | II.4 | | human | stool | 2009 | 2007 | 11 |
| 228 | Beijing | GQ856401 | 212 | II.4 | | human | stool | 2009 | 2007 | 11 |
| 229 | Beijing | GQ856402 | 212 | II.4 | | human | stool | 2009 | 2007 | 11 |
| 230 | Beijing | GQ856403 | 212 | II.4 | | human | stool | 2009 | 2007 | 11 |
| 231 | Beijing | GQ856404 | 224 | II.4 | | human | stool | 2009 | 2007 | 11 |
| 232 | Beijing | GQ856405 | 212 | II.4 | | human | stool | 2009 | 2007 | 11 |
| 233 | Beijing | GQ856406 | 212 | II.4 | | human | stool | 2009 | 2007 | 11 |
| 234 | Beijing | GQ856407 | 212 | II.4 | | human | stool | 2009 | 2007 | 11 |
| 235 | Beijing | GQ856408 | 214 | II.4 | | human | stool | 2009 | 2007 | 11 |
| 236 | Beijing | GQ856409 | 212 | II.4 | | human | stool | 2009 | 2007 | 11 |
| 237 | Beijing | GQ856410 | 212 | II.4 | | human | stool | 2009 | 2007 | 11 |
| 238 | Beijing | GQ856411 | 247 | II.4 | | human | stool | 2009 | 2007 | 11 |
| 239 | Beijing | GQ856412 | 247 | II.4 | | human | stool | 2009 | 2007 | 11 |
| 240 | Beijing | GQ856413 | 232 | II.4 | | human | stool | 2009 | 2007 | 11 |
| 241 | Beijing | GQ856414 | 240 | II.4 | | human | stool | 2009 | 2007 | 11 |
| 242 | Beijing | GQ856415 | 231 | II.4 | | human | stool | 2009 | 2007 | 11 |
| 243 | Beijing | GQ856416 | 231 | II.4 | | human | stool | 2009 | 2007 | 11 |
| 244 | Beijing | GQ856417 | 232 | II.4 | | human | stool | 2009 | 2007 | 11 |
| 245 | Beijing | GQ856418 | 247 | II.4 | | human | stool | 2009 | 2007 | 11 |
| 246 | Beijing | GQ856419 | 230 | II.4 | | human | stool | 2009 | 2007 | 11 |
| 247 | Beijing | GQ856420 | 247 | II.4 | | human | stool | 2009 | 2007 | 11 |
| 248 | Beijing | GQ856421 | 212 | II.4 | | human | stool | 2009 | 2007 | 11 |
| 249 | Beijing | GQ856422 | 219 | II.4 | | human | stool | 2009 | 2007 | 11 |
| 250 | Beijing | GQ856423 | 214 | II.4 | | human | stool | 2009 | 2007 | 11 |
| 251 | Beijing | GQ856424 | 212 | II.4 | | human | stool | 2009 | 2007 | 11 |
| 252 | Beijing | GQ856425 | 212 | II.4 | | human | stool | 2009 | 2007 | 11 |
| 253 | Beijing | GQ856426 | 212 | II.4 | | human | stool | 2009 | 2007 | 11 |
| 254 | Beijing | GQ856427 | 212 | II.4 | | human | stool | 2009 | 2008 | 11 |

| | | | | | | | | | | |
|---|---|---|---|---|---|---|---|---|---|---|
| 255 | Beijing | GQ856428 | 247 | II.4 | | human | stool | 2009 | 2008 | 11 |
| 256 | Beijing | GQ856429 | 237 | II.4 | | human | stool | 2009 | 2008 | 11 |
| 257 | Beijing | GQ856430 | 231 | II.12 | | human | stool | 2009 | 2008 | 11 |
| 258 | Beijing | GQ856431 | 246 | II.4 | | human | stool | 2009 | 2008 | 11 |
| 259 | Beijing | GQ856432 | 247 | II.4 | | human | stool | 2009 | 2008 | 11 |
| 260 | Beijing | GQ856433 | 233 | II.4 | | human | stool | 2009 | 2008 | 11 |
| 261 | Beijing | GQ856434 | 240 | II.4 | | human | stool | 2009 | 2008 | 11 |
| 262 | Beijing | GQ856435 | 240 | II.4 | | human | stool | 2009 | 2008 | 11 |
| 263 | Beijing | GQ856436 | 219 | II.4 | | human | stool | 2009 | 2008 | 11 |
| 264 | Beijing | GQ856437 | 247 | II.4 | | human | stool | 2009 | 2008 | 11 |
| 265 | Beijing | GQ856438 | 247 | II.4 | | human | stool | 2009 | 2008 | 11 |
| 266 | Beijing | GQ856439 | 247 | II.4 | | human | stool | 2009 | 2008 | 11 |
| 267 | Beijing | GQ856440 | 247 | II.4 | | human | stool | 2009 | 2008 | 11 |
| 268 | Beijing | GQ856441 | 243 | II.4 | | human | stool | 2009 | 2008 | 11 |
| 269 | Beijing | GQ856443 | 256 | II.15 | | human | stool | 2009 | 2007 | 11 |
| 270 | Beijing | GQ856445 | 3267 | II.4 | II.4 | human | stool | 2009 | 2007 | 11 |
| 271 | Beijing | GQ856446 | 3267 | II.4 | II.4 | human | stool | 2009 | 2007 | 11 |
| 272 | Beijing | GQ856447 | 3267 | II.4 | II.4 | human | stool | 2009 | 2007 | 11 |
| 273 | Beijing | GQ856448 | 3267 | II.4 | II.4 | human | stool | 2009 | 2007 | 11 |
| 274 | Beijing | GQ856449 | 3267 | II.4 | II.4 | human | stool | 2009 | 2007 | 11 |
| 275 | Beijing | GQ856450 | 3267 | II.4 | II.4 | human | stool | 2009 | 2007 | 11 |
| 276 | Beijing | GQ856451 | 3267 | II.4 | II.4 | human | stool | 2009 | 2007 | 11 |
| 277 | Beijing | GQ856452 | 3267 | II.4 | II.4 | human | stool | 2009 | 2007 | 11 |
| 278 | Beijing | GQ856453 | 3267 | II.4 | II.4 | human | stool | 2009 | 2007 | 11 |
| 279 | Beijing | GQ856454 | 3267 | II.4 | II.4 | human | stool | 2009 | 2007 | 11 |
| 280 | Beijing | GQ856455 | 3267 | II.4 | II.4 | human | stool | 2009 | 2007 | 11 |
| 281 | Beijing | GQ856456 | 3267 | II.4 | II.4 | human | stool | 2009 | 2007 | 11 |
| 282 | Beijing | GQ856457 | 3267 | II.4 | II.4 | human | stool | 2009 | 2008 | 11 |
| 283 | Beijing | GQ856458 | 3267 | II.4 | II.4 | human | stool | 2009 | 2008 | 11 |
| 284 | Beijing | GQ856459 | 3267 | II.4 | II.4 | human | stool | 2009 | 2008 | 11 |
| 285 | Beijing | GQ856460 | 3267 | II.4 | II.4 | human | stool | 2009 | 2008 | 11 |
| 286 | Beijing | GQ856461 | 3267 | II.4 | II.4 | human | stool | 2009 | 2007 | 11 |
| 287 | Beijing | GQ856462 | 3120 | I.8 | I.8 | human | stool | 2009 | 2007 | 11 |
| 288 | Beijing | GQ856463 | 3109 | I.b | I.6 | human | stool | 2009 | 2007 | 11 |
| 289 | Beijing | GQ856464 | 3109 | I.b | I.6 | human | stool | 2009 | 2007 | 11 |
| 290 | Beijing | GQ856465 | 3237 | II.7 | II.14 | human | stool | 2009 | 2007 | 11 |
| 291 | Beijing | GQ856466 | 3230 | II.12 | II.3 | human | stool | 2009 | 2007 | 11 |
| 292 | Beijing | GQ856467 | 3230 | II.12 | II.3 | human | stool | 2009 | 2007 | 11 |
| 293 | Beijing | GQ856468 | 3174 | II.b | II.21 | human | stool | 2009 | 2008 | 11 |
| 294 | Beijing | GQ856469 | 3061 | II.n | II.22 | human | stool | 2009 | 2007 | 11 |
| 295 | Beijing | GQ856470 | 3084 | I.d | I.3 | human | stool | 2009 | 2007 | 11 |
| 296 | Beijing | GQ856471 | 3132 | I.d | I.3 | human | stool | 2009 | 2007 | 11 |
| 297 | Beijing | GQ856472 | 3132 | I.d | I.3 | human | stool | 2009 | 2007 | 11 |

| | | | | | | | | | | |
|---|---|---|---|---|---|---|---|---|---|---|
| 298 | Beijing | GQ856473 | 3173 | I.a | I.3 | human | stool | 2009 | 2007 | 11 |
| 299 | Beijing | GQ856474 | 3249 | II.15 | II.15 | human | stool | 2009 | 2008 | 11 |
| 300 | Beijing | GQ856475 | 3119 | I.4 | I.4 | human | stool | 2009 | 2008 | 11 |
| 301 | Beijing | GQ856476 | 3246 | II.16 | II.16 | human | stool | 2009 | 2008 | 11 |
| 302 | Beijing | GU350225 | 1623 | | II.4 | human | unclear | 2009 | 2005 | unpublished |
| 303 | Beijing | HM991362 | 233 | II.4 | | human | stool | 2010 | 2007 | 15 |
| 304 | Beijing | HM991363 | 233 | II.4 | | human | stool | 2010 | 2007 | 15 |
| 305 | Beijing | HM991364 | 233 | II.4 | | human | stool | 2010 | 2007 | 15 |
| 306 | Beijing | HM991365 | 233 | II.4 | | human | stool | 2010 | 2007 | 15 |
| 307 | Beijing | HM991366 | 233 | II.4 | | human | stool | 2010 | 2007 | 15 |
| 308 | Beijing | HM991367 | 233 | II.4 | | human | stool | 2010 | 2007 | 15 |
| 309 | Beijing | HM991368 | 260 | II.4 | | human | stool | 2010 | 2007 | 15 |
| 310 | Beijing | HM991369 | 273 | II.4 | | human | stool | 2010 | 2007 | 15 |
| 311 | Beijing | HM991370 | 269 | I.3 | | human | stool | 2010 | 2007 | 15 |
| 312 | Beijing | HM991371 | 273 | II.4 | | human | stool | 2010 | 2007 | 15 |
| 313 | Beijing | HM991372 | 273 | II.4 | | human | stool | 2010 | 2007 | 15 |
| 314 | Beijing | HM991373 | 273 | II.4 | | human | stool | 2010 | 2007 | 15 |
| 315 | Beijing | HM991374 | 233 | II.4 | | human | stool | 2010 | 2007 | 15 |
| 316 | Beijing | HM991375 | 233 | II.4 | | human | stool | 2010 | 2007 | 15 |
| 317 | Beijing | HM991376 | 233 | II.12 | | human | stool | 2010 | 2007 | 15 |
| 318 | Beijing | HM991377 | 273 | II.4 | | human | stool | 2010 | 2007 | 15 |
| 319 | Beijing | HM991378 | 233 | II.4 | | human | stool | 2010 | 2007 | 15 |
| 320 | Beijing | HM991379 | 233 | II.4 | | human | stool | 2010 | 2007 | 15 |
| 321 | Beijing | HM991380 | 233 | II.b | | human | stool | 2010 | 2007 | 15 |
| 322 | Beijing | HM991381 | 233 | II.4 | | human | stool | 2010 | 2007 | 15 |
| 323 | Beijing | HM991382 | 233 | II.4 | | human | stool | 2010 | 2007 | 15 |
| 324 | Beijing | HM991383 | 233 | II.4 | | human | stool | 2010 | 2007 | 15 |
| 325 | Beijing | HM991384 | 233 | II.4 | | human | stool | 2010 | 2007 | 15 |
| 326 | Beijing | HM991385 | 233 | II.b | | human | stool | 2010 | 2007 | 15 |
| 327 | Beijing | HM991386 | 233 | II.12 | | human | stool | 2010 | 2007 | 15 |
| 328 | Beijing | HM991387 | 233 | II.4 | | human | stool | 2010 | 2007 | 15 |
| 329 | Beijing | HM991388 | 233 | II.4 | | human | stool | 2010 | 2008 | 15 |
| 330 | Beijing | HM991389 | 233 | II.4 | | human | stool | 2010 | 2008 | 15 |
| 331 | Beijing | HM991390 | 261 | II.4 | | human | stool | 2010 | 2008 | 15 |
| 332 | Beijing | HM991391 | 233 | II.12 | | human | stool | 2010 | 2008 | 15 |
| 333 | Beijing | HM991392 | 233 | II.4 | | human | stool | 2010 | 2008 | 15 |
| 334 | Beijing | HM991393 | 233 | II.4 | | human | stool | 2010 | 2008 | 15 |
| 335 | Beijing | HM991394 | 233 | II.4 | | human | stool | 2010 | 2008 | 15 |
| 336 | Beijing | HM991395 | 233 | II.4 | | human | stool | 2010 | 2008 | 15 |
| 337 | Beijing | HM991396 | 233 | II.4 | | human | stool | 2010 | 2008 | 15 |
| 338 | Beijing | HM991397 | 233 | II.4 | | human | stool | 2010 | 2008 | 15 |
| 339 | Beijing | HM991398 | 273 | II.4 | | human | stool | 2010 | 2008 | 15 |
| 340 | Beijing | HM991399 | 273 | II.7 | | human | stool | 2010 | 2008 | 15 |

| | | | | | | | | | | |
|---|---|---|---|---|---|---|---|---|---|---|
| 341 | Beijing | HM991400 | 233 | II.4 | | human | stool | 2010 | 2008 | 15 |
| 342 | Beijing | HM991401 | 233 | II.4 | | human | stool | 2010 | 2008 | 15 |
| 343 | Beijing | HM991403 | 233 | II.4 | | human | stool | 2010 | 2008 | 15 |
| 344 | Beijing | HM991404 | 253 | II.4 | | human | stool | 2010 | 2008 | 15 |
| 345 | Beijing | HM991405 | 273 | II.4 | | human | stool | 2010 | 2007 | 15 |
| 346 | Beijing | HM991406 | 273 | I.4 | | human | stool | 2010 | 2008 | 15 |
| 347 | Beijing | HM991407 | 273 | II.4 | | human | stool | 2010 | 2008 | 15 |
| 348 | Beijing | HM991408 | 233 | I.b | | human | stool | 2010 | 2008 | 15 |
| 349 | Beijing | HM991409 | 273 | II.b | | human | stool | 2010 | 2008 | 15 |
| 350 | Beijing | HM991410 | 281 | | II.4 | human | stool | 2010 | 2007 | 15 |
| 351 | Beijing | HM991411 | 247 | | II.4 | human | stool | 2010 | 2007 | 15 |
| 352 | Beijing | HM991412 | 281 | | II.4 | human | stool | 2010 | 2007 | 15 |
| 353 | Beijing | HM991413 | 247 | | II.4 | human | stool | 2010 | 2007 | 15 |
| 354 | Beijing | HM991414 | 281 | | II.4 | human | stool | 2010 | 2007 | 15 |
| 355 | Beijing | HM991415 | 247 | | II.4 | human | stool | 2010 | 2007 | 15 |
| 356 | Beijing | HM991416 | 261 | | I.3 | human | stool | 2010 | 2008 | 15 |
| 357 | Beijing | HM991417 | 247 | | II.4 | human | stool | 2010 | 2007 | 15 |
| 358 | Beijing | HM991418 | 247 | | II.4 | human | stool | 2010 | 2007 | 15 |
| 359 | Beijing | HM991419 | 222 | | II.3 | human | stool | 2010 | 2007 | 15 |
| 360 | Beijing | HM991420 | 247 | | II.4 | human | stool | 2010 | 2007 | 15 |
| 361 | Beijing | HM991421 | 281 | | II.4 | human | stool | 2010 | 2007 | 15 |
| 362 | Beijing | HM991422 | 281 | | II.4 | human | stool | 2010 | 2007 | 15 |
| 363 | Beijing | HM991423 | 281 | | II.21 | human | stool | 2010 | 2007 | 15 |
| 364 | Beijing | HM991424 | 281 | | II.4 | human | stool | 2010 | 2007 | 15 |
| 365 | Beijing | HM991425 | 281 | | II.4 | human | stool | 2010 | 2007 | 15 |
| 366 | Beijing | HM991426 | 281 | | II.4 | human | stool | 2010 | 2007 | 15 |
| 367 | Beijing | HM991427 | 281 | | II.4 | human | stool | 2010 | 2007 | 15 |
| 368 | Beijing | HM991428 | 281 | | II.21 | human | stool | 2010 | 2007 | 15 |
| 369 | Beijing | HM991429 | 281 | | II.3 | human | stool | 2010 | 2007 | 15 |
| 370 | Beijing | HM991430 | 247 | | II.4 | human | stool | 2010 | 2008 | 15 |
| 371 | Beijing | HM991431 | 247 | | II.4 | human | stool | 2010 | 2007 | 15 |
| 372 | Beijing | HM991432 | 247 | | II.4 | human | stool | 2010 | 2008 | 15 |
| 373 | Beijing | HM991433 | 247 | | II.4 | human | stool | 2010 | 2008 | 15 |
| 374 | Beijing | HM991434 | 281 | | II.4 | human | stool | 2010 | 2008 | 15 |
| 375 | Beijing | HM991435 | 247 | | II.4 | human | stool | 2010 | 2008 | 15 |
| 376 | Beijing | HM991436 | 281 | | II.4 | human | stool | 2010 | 2008 | 15 |
| 377 | Beijing | HM991437 | 281 | | II.4 | human | stool | 2010 | 2008 | 15 |
| 378 | Beijing | HM991438 | 281 | | II.4 | human | stool | 2010 | 2008 | 15 |
| 379 | Beijing | HM991439 | 281 | | II.14 | human | stool | 2010 | 2008 | 15 |
| 380 | Beijing | HM991440 | 281 | | II.4 | human | stool | 2010 | 2008 | 15 |
| 381 | Beijing | HM991441 | 247 | | II.2 | human | stool | 2010 | 2008 | 15 |
| 382 | Beijing | HM991442 | 261 | | I.7 | human | stool | 2010 | 2008 | 15 |
| 383 | Beijing | HM991443 | 281 | | II.4 | human | stool | 2010 | 2008 | 15 |

| | | | | | | | | | | |
|---|---|---|---|---|---|---|---|---|---|---|
| 384 | Beijing | HM991444 | 281 | | II.4 | human | stool | 2010 | 2008 | 15 |
| 385 | Beijing | HM991445 | 231 | | I.4 | human | stool | 2010 | 2008 | 15 |
| 386 | Beijing | HM991446 | 247 | | II.4 | human | stool | 2010 | 2008 | 15 |
| 387 | Beijing | HM991447 | 261 | | I.6 | human | stool | 2010 | 2008 | 15 |
| 388 | Beijing | HM991448 | 281 | | II.21 | human | stool | 2010 | 2008 | 15 |
| 389 | Beijing | HQ609493 | 273 | | II.4 | human | stool | 2010 | 2007 | 15 |
| 390 | Beijing | HQ609494 | 281 | | II.4 | human | stool | 2010 | 2008 | 15 |
| 391 | Beijing | HQ609495 | 281 | | II.3 | human | stool | 2010 | 2008 | 15 |
| 392 | Beijing | HQ609496 | 281 | | II.4 | human | stool | 2010 | 2008 | 15 |
| 393 | Beijing | HQ609497 | 272 | | II.4 | human | stool | 2010 | 2007 | 15 |
| 394 | Beijing | HQ609498 | 281 | | II.4 | human | stool | 2010 | 2007 | 15 |
| 395 | Beijing | JQ889812 | 1009 | II.g | II.12 | human | unclear | 2012 | 2009 | unpublished |
| 396 | Beijing | JQ889814 | 1009 | II.g | II.12 | human | unclear | 2012 | 2009 | unpublished |
| 397 | Beijing | JQ889815 | 1009 | II.g | II.12 | human | unclear | 2012 | 2009 | unpublished |
| 398 | Beijing | JQ889816 | 1009 | II.7 | II.6 | human | unclear | 2012 | 2011 | unpublished |
| 399 | Beijing | JQ889817 | 1009 | II.16 | II.2 | human | unclear | 2012 | 2011 | unpublished |
| 400 | Beijing | JQ899442 | 1009 | II.g | II.12 | human | unclear | 2012 | 2010 | unpublished |
| 401 | Chongqing | JN596789 | 282 | | II.7 | human | stool | 2011 | 2009 | 16 |
| 402 | Chongqing | JN596791 | 282 | | II.12 | human | stool | 2011 | 2009 | 16 |
| 403 | Chongqing | JN596794 | 282 | | II.2 | human | stool | 2011 | 2009 | 16 |
| 404 | Chongqing | JN596796 | 282 | | II.13 | human | stool | 2011 | 2009 | 16 |
| 405 | Chongqing | JN596798 | 282 | | II.4 | human | stool | 2011 | 2009 | 16 |
| 406 | Chongqing | JN596804 | 282 | | II.4 | human | stool | 2011 | 2008 | 16 |
| 407 | Chongqing | JN596807 | 282 | | II.6 | human | stool | 2011 | 2009 | 16 |
| 408 | Chongqing | JN596813 | 282 | | II.3 | human | stool | 2011 | 2009 | 16 |
| 409 | Fujian | EF670624 | 281 | | II.4 | human | stool | 2007 | 2006 | 1 |
| 410 | Fujian | EF670625 | 281 | | II.4 | human | stool | 2007 | 2006 | 1 |
| 411 | Fujian | EF670626 | 281 | | II.4 | human | stool | 2007 | 2006 | 1 |
| 412 | Fujian | EF670641 | 281 | | II.4 | human | stool | 2007 | 2006 | 1 |
| 413 | Fujian | EF670642 | 281 | | II.4 | human | stool | 2007 | 2006 | 1 |
| 414 | Fujian | EF670643 | 281 | | II.4 | human | stool | 2007 | 2006 | 1 |
| 415 | Fujian | EF670644 | 281 | | II.4 | human | stool | 2007 | 2006 | 1 |
| 416 | Fujian | EU072286 | 274 | II.4 | | human | unclear | 2007 | 1999~2005 | unpublished |
| 417 | Fujian | EU072318 | 274 | II.4 | | human | unclear | 2007 | 1999~2005 | unpublished |
| 418 | Fujian | EU072352 | 274 | II.a | | human | unclear | 2007 | 1999~2005 | unpublished |
| 419 | Fujian | HM195192 | 324 | | II.4 | human | unclear | 2010 | 2010 | unpublished |
| 420 | Fujian | HM195193 | 324 | | II.4 | human | unclear | 2010 | 2010 | unpublished |
| 421 | Fujian | HM195194 | 324 | | II.4 | human | unclear | 2010 | 2010 | unpublished |
| 422 | Fujian | HM195195 | 326 | | II.4 | human | unclear | 2010 | 2010 | unpublished |
| 423 | Fujian | HM195196 | 242 | | I.6 | human | unclear | 2010 | 2010 | unpublished |
| 424 | Fujian | HM195197 | 243 | | I.6 | human | unclear | 2010 | 2010 | unpublished |
| 425 | Fujian | HM195199 | 328 | II.4 | | human | unclear | 2010 | 2010 | unpublished |
| 426 | Fujian | HM195200 | 329 | II.4 | | human | unclear | 2010 | 2010 | unpublished |

| | | | | | | | | | | |
|---|---|---|---|---|---|---|---|---|---|---|
| 427 | Fujian | JF947180 | 306 | | II.4 | human | unclear | 2011 | 2011 | unpublished |
| 428 | Fujian | JF947181 | 306 | | II.6 | human | unclear | 2011 | 2011 | unpublished |
| 429 | Gansu | DQ364459 | 3120 | II.4 | II.4 | human | unclear | 2006 | 2002 | unpublished |
| 430 | Gansu | EU072255 | 274 | II.3 | | human | unclear | 2007 | 1999~2005 | unpublished |
| 431 | Gansu | EU072256 | 274 | II.12 | | human | unclear | 2007 | 1999~2005 | unpublished |
| 432 | Gansu | EU072257 | 274 | II.12 | | human | unclear | 2007 | 1999~2005 | unpublished |
| 433 | Gansu | EU072258 | 274 | II.12 | | human | unclear | 2007 | 1999~2005 | unpublished |
| 434 | Gansu | EU072259 | 274 | II.12 | | human | unclear | 2007 | 1999~2005 | unpublished |
| 435 | Gansu | EU072260 | 274 | II.12 | | human | unclear | 2007 | 1999~2005 | unpublished |
| 436 | Gansu | EU072261 | 274 | II.12 | | human | unclear | 2007 | 1999~2005 | unpublished |
| 437 | Gansu | EU072262 | 274 | II.12 | | human | unclear | 2007 | 1999~2005 | unpublished |
| 438 | Gansu | EU072263 | 274 | II.12 | | human | unclear | 2007 | 1999~2005 | unpublished |
| 439 | Gansu | EU072264 | 274 | II.12 | | human | unclear | 2007 | 1999~2005 | unpublished |
| 440 | Gansu | EU072265 | 274 | II.12 | | human | unclear | 2007 | 1999~2005 | unpublished |
| 441 | Gansu | EU072266 | 274 | II.12 | | human | unclear | 2007 | 1999~2005 | unpublished |
| 442 | Gansu | EU072267 | 274 | II.3 | | human | unclear | 2007 | 1999~2005 | unpublished |
| 443 | Gansu | EU072268 | 274 | II.12 | | human | unclear | 2007 | 1999~2005 | unpublished |
| 444 | Gansu | EU072269 | 274 | II.12 | | human | unclear | 2007 | 1999~2005 | unpublished |
| 445 | Gansu | EU072270 | 274 | II.3 | | human | unclear | 2007 | 1999~2005 | unpublished |
| 446 | Gansu | EU072271 | 274 | II.3 | | human | unclear | 2007 | 1999~2005 | unpublished |
| 447 | Gansu | EU072272 | 274 | II.12 | | human | unclear | 2007 | 1999~2005 | unpublished |
| 448 | Gansu | EU072273 | 274 | II.7 | | human | unclear | 2007 | 1999~2005 | unpublished |
| 449 | Gansu | EU072274 | 274 | II.12 | | human | unclear | 2007 | 1999~2005 | unpublished |
| 450 | Gansu | EU072275 | 274 | II.3 | | human | unclear | 2007 | 1999~2005 | unpublished |
| 451 | Gansu | EU072276 | 274 | II.12 | | human | unclear | 2007 | 1999~2005 | unpublished |
| 452 | Gansu | EU072280 | 274 | II.12 | | human | unclear | 2007 | 1999~2005 | unpublished |
| 453 | Gansu | EU072281 | 274 | II.4 | | human | unclear | 2007 | 1999~2005 | unpublished |
| 454 | Guangdong | DQ369797 | 7558 | II.12 | II.4 | human | unclear | 2006 | unclear | unpublished |
| 455 | Guangdong | EF535854 | 1623 | | II.4 | human | stool | 2007 | 2006 | unpublished |
| 456 | Guangdong | EU794696 | 1324 | II.4 | | human | stool | 2008 | 2006 | unpublished |
| 457 | Guangdong | EU794697 | 1399 | II.4 | | human | stool | 2008 | 2006 | unpublished |
| 458 | Guangdong | EU794698 | 1422 | II.12 | | human | stool | 2008 | 2006 | unpublished |
| 459 | Guangdong | EU794699 | 1317 | II.12 | | human | stool | 2008 | 2006 | unpublished |
| 460 | Guangdong | EU794700 | 1322 | II.4 | | human | stool | 2008 | 2006 | unpublished |
| 461 | Guangdong | EU794701 | 1333 | II.12 | | human | stool | 2008 | 2006 | unpublished |
| 462 | Guangdong | EU794702 | 1322 | II.4 | | human | stool | 2008 | 2006 | unpublished |
| 463 | Guangdong | EU794703 | 1323 | II.4 | | human | stool | 2008 | 2006 | unpublished |
| 464 | Guangdong | EU794704 | 1323 | II.4 | | human | stool | 2008 | 2006 | unpublished |
| 465 | Guangdong | EU794705 | 1402 | II.12 | | human | stool | 2008 | 2006 | unpublished |
| 466 | Guangdong | EU794706 | 1410 | II.12 | | human | stool | 2008 | 2006 | unpublished |
| 467 | Guangdong | EU794707 | 1335 | II.4 | | human | stool | 2008 | 2006 | unpublished |
| 468 | Guangdong | EU794708 | 1332 | II.4 | | human | stool | 2008 | 2006 | unpublished |
| 469 | Guangdong | EU794709 | 1332 | II.4 | | human | stool | 2008 | 2006 | unpublished |

| | | | | | | | | | | |
|---|---|---|---|---|---|---|---|---|---|---|
| 470 | Guangdong | EU794710 | 1336 | II.4 | | human | stool | 2008 | 2006 | unpublished |
| 471 | Guangdong | EU794711 | 1335 | II.4 | | human | stool | 2008 | 2006 | unpublished |
| 472 | Guangdong | EU794712 | 1325 | II.4 | | human | stool | 2008 | 2006 | unpublished |
| 473 | Guangdong | EU794713 | 1315 | II.4 | | human | stool | 2008 | 2006 | unpublished |
| 474 | Guangdong | EU794714 | 1333 | II.12 | | human | stool | 2008 | 2006 | unpublished |
| 475 | Guangdong | EU794715 | 1326 | II.4 | | human | stool | 2008 | 2006 | unpublished |
| 476 | Guangdong | EU794716 | 1336 | II.12 | | human | stool | 2008 | 2006 | unpublished |
| 477 | Guangdong | EU794717 | 1340 | II.12 | | human | stool | 2008 | 2006 | unpublished |
| 478 | Guangdong | GQ223399 | 279 | | II.4 | human | stool | 2009 | 2007 | 6 |
| 479 | Guangdong | GQ223400 | 279 | | II.4 | human | stool | 2009 | 2007 | 6 |
| 480 | Guangdong | GQ223401 | 279 | | II.4 | human | stool | 2009 | 2007 | 6 |
| 481 | Guangdong | GQ223402 | 279 | | II.4 | human | stool | 2009 | 2007 | 6 |
| 482 | Guangdong | GQ223403 | 279 | | II.4 | human | stool | 2009 | 2007 | 6 |
| 483 | Guangdong | GQ223404 | 279 | | II.4 | human | stool | 2009 | 2007 | 6 |
| 484 | Guangdong | HM627865 | 344 | | II.4 | human | stool | 2010 | 2009 | unpublished |
| 485 | Guangdong | HM627866 | 344 | | II.4 | human | stool | 2010 | 2009 | unpublished |
| 486 | Guangdong | HM627867 | 344 | | II.4 | human | stool | 2010 | 2009 | unpublished |
| 487 | Guangdong | HM627868 | 344 | | II.4 | human | stool | 2010 | 2009 | unpublished |
| 488 | Guangdong | HM627869 | 344 | | II.4 | human | stool | 2010 | 2009 | unpublished |
| 489 | Guangdong | HM627870 | 262 | II.4 | | human | stool | 2010 | 2009 | unpublished |
| 490 | Guangdong | HM627871 | 262 | II.4 | | human | stool | 2010 | 2009 | unpublished |
| 491 | Guangdong | HM627872 | 262 | II.4 | | human | stool | 2010 | 2009 | unpublished |
| 492 | Guangdong | HM627873 | 262 | II.4 | | human | stool | 2010 | 2009 | unpublished |
| 493 | Guangdong | HM627874 | 262 | II.4 | | human | stool | 2010 | 2009 | unpublished |
| 494 | Guangdong | JQ861907 | 580 | II.4 | II.4 | human | stool | 2012 | 2011 | unpublished |
| 495 | Guangdong | JQ861908 | 583 | II.12 | II.3 | human | stool | 2012 | 2011 | unpublished |
| 496 | Guangdong | JQ861909 | 583 | II.4 | II.4 | human | stool | 2012 | 2011 | unpublished |
| 497 | Guangdong | JQ861910 | 583 | II.4 | II.4 | human | stool | 2012 | 2011 | unpublished |
| 498 | Guangdong | JQ861911 | 583 | II.4 | II.4 | human | stool | 2012 | 2011 | unpublished |
| 499 | Guangdong | JQ861912 | 583 | II.4 | II.4 | human | stool | 2012 | 2011 | unpublished |
| 500 | Guangdong | JQ861913 | 583 | II.4 | II.4 | human | stool | 2012 | 2011 | unpublished |
| 501 | Guangdong | JQ861914 | 583 | II.4 | II.4 | human | stool | 2012 | 2011 | unpublished |
| 502 | Guangdong | JQ861915 | 583 | II.4 | II.4 | human | stool | 2012 | 2011 | unpublished |
| 503 | Guangdong | JQ861916 | 583 | II.4 | II.4 | human | stool | 2012 | 2011 | unpublished |
| 504 | Guangdong | JQ861917 | 583 | II.4 | II.4 | human | stool | 2012 | 2011 | unpublished |
| 505 | Guangdong | JQ861918 | 583 | II.4 | II.4 | human | stool | 2012 | 2011 | unpublished |
| 506 | Guangdong | JQ861919 | 583 | II.4 | II.4 | human | stool | 2012 | 2011 | unpublished |
| 507 | Guangdong | JQ861920 | 583 | II.4 | II.4 | human | stool | 2012 | 2011 | unpublished |
| 508 | Guangdong | JQ861921 | 583 | II.4 | II.4 | human | stool | 2012 | 2011 | unpublished |
| 509 | Guangdong | JQ861922 | 583 | II.4 | II.4 | human | stool | 2012 | 2011 | unpublished |
| 510 | Guangdong | JQ861923 | 583 | II.4 | II.4 | human | stool | 2012 | 2011 | unpublished |
| 511 | Guangdong | JQ861924 | 583 | II.4 | II.4 | human | stool | 2012 | 2011 | unpublished |
| 512 | Guangdong | JQ861925 | 583 | II.4 | II.4 | human | stool | 2012 | 2011 | unpublished |

| | | | | | | | | | | |
|---|---|---|---|---|---|---|---|---|---|---|
| 513 | Guangdong | JQ861926 | 583 | II.4 | II.4 | human | stool | 2012 | 2011 | unpublished |
| 514 | Guangdong | JQ861927 | 583 | II.4 | II.4 | human | stool | 2012 | 2011 | unpublished |
| 515 | Guangdong | JQ861928 | 583 | II.4 | II.4 | human | stool | 2012 | 2011 | unpublished |
| 516 | Guangdong | JQ861929 | 583 | II.4 | II.4 | human | stool | 2012 | 2011 | unpublished |
| 517 | Guangdong | JQ861930 | 583 | II.4 | II.4 | human | stool | 2012 | 2011 | unpublished |
| 518 | Guangdong | JQ861931 | 583 | II.4 | II.4 | human | stool | 2012 | 2011 | unpublished |
| 519 | Guangdong | JQ861932 | 583 | II.4 | II.4 | human | stool | 2012 | 2011 | unpublished |
| 520 | Guangxi | EU408325 | 282 | | II.4 | human | stool | 2008 | 2006 | unpublished |
| 521 | Guangxi | GU186913 | 595 | I.b | I.6 | human | stool | 2009 | 2007 | unpublished |
| 522 | Guangxi | HM104664 | 474 | II.12 | II.12 | human | stool | 2010 | 2007 | unpublished |
| 523 | Guangxi | HM104665 | 387 | | II.21 | human | stool | 2010 | 2007 | unpublished |
| 524 | Guangxi | HM104666 | 387 | | II.21 | human | stool | 2010 | 2007 | unpublished |
| 525 | Guangxi | HM241691 | 390 | | I.1 | human | stool | 2010 | 2010 | unpublished |
| 526 | Guizhou | EU072279 | 274 | II.4 | | human | unclear | 2007 | 1999~2005 | unpublished |
| 527 | Guizhou | EU072317 | 274 | II.4 | | human | unclear | 2007 | 1999~2005 | unpublished |
| 528 | Guizhou | EU072320 | 274 | II.4 | | human | unclear | 2007 | 1999~2005 | unpublished |
| 529 | Guizhou | JQ934807 | 364 | | II.4 | human | stool | 2012 | 2010 | unpublished |
| 530 | Guizhou | JQ934808 | 364 | | II.4 | human | stool | 2012 | 2010 | unpublished |
| 531 | Guizhou | JQ934809 | 364 | | II.4 | human | stool | 2012 | 2010 | unpublished |
| 532 | Guizhou | JQ934810 | 364 | | II.4 | human | stool | 2012 | 2010 | unpublished |
| 533 | Guizhou | JQ934811 | 364 | | II.4 | human | stool | 2012 | 2010 | unpublished |
| 534 | Guizhou | JQ934812 | 364 | | II.4 | human | stool | 2012 | 2010 | unpublished |
| 535 | Guizhou | JQ934813 | 364 | | II.4 | human | stool | 2012 | 2010 | unpublished |
| 536 | Guizhou | JQ934814 | 364 | | II.4 | human | stool | 2012 | 2010 | unpublished |
| 537 | Guizhou | JQ934815 | 364 | | II.4 | human | stool | 2012 | 2010 | unpublished |
| 538 | Guizhou | JQ934816 | 364 | | II.3 | human | stool | 2012 | 2010 | unpublished |
| 539 | Guizhou | JQ934817 | 364 | | II.3 | human | stool | 2012 | 2010 | unpublished |
| 540 | Guizhou | JQ934818 | 364 | | II.3 | human | stool | 2012 | 2010 | unpublished |
| 541 | Guizhou | JQ934819 | 364 | | II.3 | human | stool | 2012 | 2010 | unpublished |
| 542 | Guizhou | JQ934820 | 364 | | II.2 | human | stool | 2012 | 2010 | unpublished |
| 543 | Hainan | EF670590 | 292 | | I.2 | human | stool | 2007 | 2006 | 1 |
| 544 | Hainan | EF670591 | 281 | | II.3 | human | stool | 2007 | 2006 | 1 |
| 545 | Hainan | EU400328 | 281 | | II.4 | human | stool | 2008 | 2006 | 1 |
| 546 | Hainan | EU400329 | 281 | | II.4 | human | stool | 2008 | 2006 | 1 |
| 547 | Hebei | EF670592 | 281 | | II.4 | human | stool | 2007 | 2006 | 1 |
| 548 | Hebei | EF670598 | 292 | | I.2 | human | stool | 2007 | 2006 | 1 |
| 549 | Hebei | EF670599 | 281 | | II.3 | human | stool | 2007 | 2006 | 1 |
| 550 | Hebei | EF670600 | 281 | | II.3 | human | stool | 2007 | 2006 | 1 |
| 551 | Hebei | EF670601 | 281 | | II.3 | human | stool | 2007 | 2006 | 1 |
| 552 | Hebei | EF670602 | 281 | | II.3 | human | stool | 2007 | 2006 | 1 |
| 553 | Hebei | EF670603 | 281 | | II.3 | human | stool | 2007 | 2006 | 1 |
| 554 | Hebei | EF670630 | 293 | | I.2 | human | stool | 2007 | 2006 | 1 |
| 555 | Hebei | EF670631 | 281 | | II.4 | human | stool | 2007 | 2006 | 1 |

| | | | | | | | | | | |
|---|---|---|---|---|---|---|---|---|---|---|
| 556 | Hebei | EF670632 | 281 | | II.4 | human | stool | 2007 | 2006 | 1 |
| 557 | Hebei | EF670633 | 281 | | II.4 | human | stool | 2007 | 2006 | 1 |
| 558 | Hebei | EF670634 | 281 | | II.4 | human | stool | 2007 | 2006 | 1 |
| 559 | Hebei | EF670635 | 281 | | II.4 | human | stool | 2007 | 2006 | 1 |
| 560 | Hebei | EF670645 | 281 | | II.4 | human | stool | 2007 | 2006 | 1 |
| 561 | Hebei | EF670646 | 281 | | II.4 | human | stool | 2007 | 2006 | 1 |
| 562 | Hebei | EF670647 | 293 | | I.2 | human | stool | 2007 | 2006 | 1 |
| 563 | Hebei | EF670648 | 293 | | I.2 | human | stool | 2007 | 2006 | 1 |
| 564 | Hebei | EF670649 | 3230 | II.12 | II.3 | human | stool | 2007 | 2006 | 1 |
| 565 | Hebei | EU072249 | 3188 | II.b | II.1 | human | unclear | 2007 | 1999~2005 | unpublished |
| 566 | Hebei | EU400330 | 281 | | II.4 | human | stool | 2008 | 2006 | 1 |
| 567 | Hebei | EU400334 | 281 | | II.4 | human | stool | 2008 | 2006 | 1 |
| 568 | Hebei | EU400365 | 281 | | II.4 | human | stool | 2008 | 2006 | 1 |
| 569 | Hebei | EU400366 | 281 | | II.4 | human | stool | 2008 | 2006 | 1 |
| 570 | Hebei | EU400367 | 281 | | II.4 | human | stool | 2008 | 2006 | 1 |
| 571 | Hebei | EU400368 | 281 | | II.4 | human | stool | 2008 | 2006 | 1 |
| 572 | Hebei | JN864947 | 387 | | II.12 | human | unclear | 2011 | 2011 | unpublished |
| 573 | Henan | EF670608 | 281 | | II.3 | human | stool | 2007 | 2006 | 1 |
| 574 | Henan | EF670609 | 293 | | I.4 | human | stool | 2007 | 2006 | 1 |
| 575 | Henan | EU400377 | 281 | | II.3 | human | stool | 2008 | 2006 | 1 |
| 576 | Hong Kong | DQ007404 | 109 | II.4 | | human | stool | 2006 | 2004 | 2 |
| 577 | Hong Kong | DQ007405 | 109 | II.12 | | human | stool | 2006 | 2004 | 2 |
| 578 | Hong Kong | DQ522116 | 248 | | I.8 | human | stool | 2006 | 2005 | unpublished |
| 579 | Hong Kong | DQ522117 | 248 | | I.5 | human | stool | 2006 | 2005 | unpublished |
| 580 | Hong Kong | DQ522118 | 248 | | I.6 | human | stool | 2006 | 2005 | unpublished |
| 581 | Hong Kong | DQ522120 | 248 | | I.5 | human | stool | 2006 | 2005 | unpublished |
| 582 | Hong Kong | DQ522122 | 248 | | I.7 | human | stool | 2006 | 2005 | unpublished |
| 583 | Hong Kong | DQ522123 | 257 | | II.4 | human | stool | 2006 | 2005 | unpublished |
| 584 | Hong Kong | DQ522124 | 257 | | II.4 | human | stool | 2006 | 2005 | unpublished |
| 585 | Hong Kong | DQ522125 | 257 | | II.2 | human | stool | 2006 | 2005 | unpublished |
| 586 | Hong Kong | DQ522126 | 257 | | II.4 | human | stool | 2006 | 2005 | unpublished |
| 587 | Hong Kong | DQ522127 | 257 | | II.4 | human | stool | 2006 | 2005 | unpublished |
| 588 | Hong Kong | DQ522128 | 257 | | II.4 | human | stool | 2006 | 2005 | unpublished |
| 589 | Hong Kong | DQ522129 | 257 | | II.4 | human | stool | 2006 | 2005 | unpublished |
| 590 | Hong Kong | DQ522130 | 257 | | II.4 | human | stool | 2006 | 2005 | unpublished |
| 591 | Hong Kong | DQ522131 | 257 | | II.4 | human | stool | 2006 | 2005 | unpublished |
| 592 | Hong Kong | DQ522132 | 257 | | II.6 | human | stool | 2006 | 2005 | unpublished |
| 593 | Hong Kong | DQ522133 | 257 | | II.4 | human | stool | 2006 | 2005 | unpublished |
| 594 | Hong Kong | DQ522134 | 257 | | II.4 | human | stool | 2006 | 2005 | unpublished |
| 595 | Hong Kong | DQ522135 | 257 | | II.4 | human | stool | 2006 | 2005 | unpublished |
| 596 | Hong Kong | DQ522136 | 257 | | II.4 | human | stool | 2006 | 2005 | unpublished |
| 597 | Hong Kong | DQ522137 | 257 | | II.16 | human | stool | 2006 | 2005 | unpublished |
| 598 | Hong Kong | DQ522138 | 257 | | II.4 | human | stool | 2006 | 2005 | unpublished |

| | | | | | | | | | | |
|---|---|---|---|---|---|---|---|---|---|---|
| 599 | Hong Kong | DQ522139 | 257 | | II.4 | human | stool | 2006 | 2005 | unpublished |
| 600 | Hong Kong | DQ522140 | 257 | | II.4 | human | stool | 2006 | 2005 | unpublished |
| 601 | Hong Kong | DQ522141 | 257 | | II.3 | human | stool | 2006 | 2005 | unpublished |
| 602 | Hong Kong | DQ522142 | 257 | | II.4 | human | stool | 2006 | 2005 | unpublished |
| 603 | Hong Kong | DQ522143 | 257 | | II.13 | human | stool | 2006 | 2005 | unpublished |
| 604 | Hong Kong | DQ522144 | 257 | | II.6 | human | stool | 2006 | 2005 | unpublished |
| 605 | Hong Kong | DQ522145 | 257 | | II.3 | human | stool | 2006 | 2005 | unpublished |
| 606 | Hong Kong | DQ522146 | 257 | | II.14 | human | stool | 2006 | 2005 | unpublished |
| 607 | Hong Kong | DQ522147 | 257 | | II.4 | human | stool | 2006 | 2005 | unpublished |
| 608 | Hong Kong | DQ522148 | 257 | | II.5 | human | stool | 2006 | 2005 | unpublished |
| 609 | Hong Kong | DQ522149 | 257 | | II.4 | human | stool | 2006 | 2005 | unpublished |
| 610 | Hong Kong | DQ522150 | 257 | | II.21 | human | stool | 2006 | 2005 | unpublished |
| 611 | Hong Kong | DQ522151 | 257 | | II.4 | human | stool | 2006 | 2005 | unpublished |
| 612 | Hong Kong | DQ522152 | 257 | | II.4 | human | stool | 2006 | 2005 | unpublished |
| 613 | Hong Kong | DQ522153 | 257 | | II.6 | human | stool | 2006 | 2005 | unpublished |
| 614 | Hong Kong | DQ522154 | 257 | | II.4 | human | stool | 2006 | 2005 | unpublished |
| 615 | Hong Kong | DQ522155 | 257 | | II.3 | human | stool | 2006 | 2005 | unpublished |
| 616 | Hong Kong | DQ522156 | 257 | | II.4 | human | stool | 2006 | 2005 | unpublished |
| 617 | Hong Kong | DQ522157 | 257 | | II.3 | human | stool | 2006 | 2005 | unpublished |
| 618 | Hong Kong | DQ522158 | 257 | | II.3 | human | stool | 2006 | 2005 | unpublished |
| 619 | Hong Kong | GQ384446 | 300 | | II.4 | human | stool | 2009 | 2009 | unpublished |
| 620 | Hong Kong | GQ384447 | 350 | | II.4 | human | stool | 2009 | 2009 | unpublished |
| 621 | Hong Kong | GQ384448 | 350 | | II.4 | human | stool | 2009 | 2009 | unpublished |
| 622 | Hong Kong | GQ384449 | 250 | | II.4 | human | stool | 2009 | 2009 | unpublished |
| 623 | Hong Kong | GQ384450 | 350 | | II.4 | human | stool | 2009 | 2009 | unpublished |
| 624 | Hong Kong | GQ384451 | 350 | | II.4 | human | stool | 2009 | 2009 | unpublished |
| 625 | Hong Kong | GQ384452 | 350 | | II.4 | human | stool | 2009 | 2009 | unpublished |
| 626 | Hong Kong | GQ384453 | 350 | | II.4 | human | stool | 2009 | 2009 | unpublished |
| 627 | Hong Kong | GQ384454 | 250 | | II.4 | human | stool | 2009 | 2009 | unpublished |
| 628 | Hong Kong | GQ384455 | 350 | | II.4 | human | stool | 2009 | 2009 | unpublished |
| 629 | Hong Kong | GQ384456 | 350 | | II.4 | human | stool | 2009 | 2009 | unpublished |
| 630 | Hong Kong | GQ384457 | 350 | | II.4 | human | stool | 2009 | 2009 | unpublished |
| 631 | Hong Kong | GQ402166 | 350 | | II.4 | human | stool | 2009 | 2009 | unpublished |
| 632 | Hong Kong | GQ411924 | 276 | | II.4 | human | stool | 2009 | 2009 | 9 |
| 633 | Hong Kong | GQ411925 | 276 | | II.4 | human | stool | 2009 | 2009 | 9 |
| 634 | Hong Kong | GQ411926 | 276 | | II.4 | human | stool | 2009 | 2009 | 9 |
| 635 | Hong Kong | GQ411927 | 276 | | II.7 | human | stool | 2009 | 2009 | 9 |
| 636 | Hong Kong | GQ411928 | 276 | | II.4 | human | stool | 2009 | 2009 | 9 |
| 637 | Hong Kong | GQ411929 | 276 | | II.4 | human | stool | 2009 | 2009 | 9 |
| 638 | Hong Kong | GQ411930 | 276 | | II.4 | human | stool | 2009 | 2009 | 9 |
| 639 | Hong Kong | GQ411931 | 276 | | II.4 | human | stool | 2009 | 2009 | 9 |
| 640 | Hong Kong | GQ411932 | 276 | | II.4 | human | stool | 2009 | 2009 | 9 |
| 641 | Hong Kong | GQ411933 | 276 | | II.4 | human | stool | 2009 | 2009 | 9 |

| | | | | | | | | | | |
|---|---|---|---|---|---|---|---|---|---|---|
| 642 | Hong Kong | GQ411934 | 276 | | II.4 | human | stool | 2009 | 2009 | 9 |
| 643 | Hong Kong | GQ411935 | 279 | | II.15 | human | stool | 2009 | 2009 | 9 |
| 644 | Hong Kong | GQ411936 | 276 | | II.4 | human | stool | 2009 | 2009 | 9 |
| 645 | Hong Kong | GQ411937 | 276 | | II.13 | human | stool | 2009 | 2009 | 9 |
| 646 | Hong Kong | GQ411938 | 276 | | II.4 | human | stool | 2009 | 2009 | 9 |
| 647 | Hong Kong | GQ411939 | 276 | | II.4 | human | stool | 2009 | 2009 | 9 |
| 648 | Hong Kong | GQ411940 | 276 | | II.4 | human | stool | 2009 | 2009 | 9 |
| 649 | Hong Kong | GQ411941 | 276 | | II.12 | human | stool | 2009 | 2009 | 9 |
| 650 | Hong Kong | GQ411942 | 276 | | II.4 | human | stool | 2009 | 2009 | 9 |
| 651 | Hong Kong | GQ411943 | 276 | | II.4 | human | stool | 2009 | 2009 | 9 |
| 652 | Hong Kong | GQ411944 | 276 | | II.4 | human | stool | 2009 | 2009 | 9 |
| 653 | Hong Kong | GQ411945 | 276 | | II.12 | human | stool | 2009 | 2009 | 9 |
| 654 | Hong Kong | GQ411946 | 276 | | II.4 | human | stool | 2009 | 2009 | 9 |
| 655 | Hong Kong | GQ411947 | 276 | | II.4 | human | stool | 2009 | 2009 | 9 |
| 656 | Hong Kong | GQ411948 | 276 | | II.4 | human | stool | 2009 | 2009 | 9 |
| 657 | Hong Kong | GQ411949 | 276 | | II.4 | human | stool | 2009 | 2009 | 9 |
| 658 | Hong Kong | GQ411950 | 276 | | II.4 | human | stool | 2009 | 2009 | 9 |
| 659 | Hong Kong | GQ411951 | 276 | | II.4 | human | stool | 2009 | 2009 | 9 |
| 660 | Hong Kong | GQ411952 | 276 | | II.4 | human | stool | 2009 | 2009 | 9 |
| 661 | Hong Kong | GQ411953 | 276 | | II.4 | human | stool | 2009 | 2009 | 9 |
| 662 | Hong Kong | GQ411954 | 276 | | II.4 | human | stool | 2009 | 2009 | 9 |
| 663 | Hong Kong | GQ411955 | 276 | | II.4 | human | stool | 2009 | 2009 | 9 |
| 664 | Hong Kong | GQ411956 | 276 | | II.13 | human | stool | 2009 | 2009 | 9 |
| 665 | Hong Kong | GQ411957 | 276 | | II.4 | human | stool | 2009 | 2009 | 9 |
| 666 | Hong Kong | GQ411958 | 276 | | II.4 | human | stool | 2009 | 2009 | 9 |
| 667 | Hong Kong | GQ411959 | 276 | | II.4 | human | stool | 2009 | 2009 | 9 |
| 668 | Hong Kong | GQ411960 | 276 | | II.4 | human | stool | 2009 | 2009 | 9 |
| 669 | Hong Kong | GQ411961 | 276 | | II.4 | human | stool | 2009 | 2009 | 9 |
| 670 | Hong Kong | GQ411962 | 276 | | II.4 | human | stool | 2009 | 2009 | 9 |
| 671 | Hong Kong | GQ411963 | 276 | | II.4 | human | stool | 2009 | 2009 | 9 |
| 672 | Hong Kong | GQ411964 | 276 | | II.4 | human | stool | 2009 | 2009 | 9 |
| 673 | Hong Kong | GQ411965 | 276 | | II.4 | human | stool | 2009 | 2009 | 9 |
| 674 | Hong Kong | GQ411966 | 276 | | II.4 | human | stool | 2009 | 2009 | 9 |
| 675 | Hong Kong | GQ411967 | 276 | | II.4 | human | stool | 2009 | 2009 | 9 |
| 676 | Hong Kong | GQ411968 | 276 | | II.3 | human | stool | 2009 | 2009 | 9 |
| 677 | Hong Kong | GQ411969 | 276 | | II.4 | human | stool | 2009 | 2009 | 9 |
| 678 | Hong Kong | GQ411970 | 276 | | II.4 | human | stool | 2009 | 2009 | 9 |
| 679 | Hong Kong | GQ411971 | 276 | | II.4 | human | stool | 2009 | 2009 | 9 |
| 680 | Hong Kong | GU228569 | 162 | II.4 | | human | stool | 2009 | 2006 | 12 |
| 681 | Hong Kong | GU228570 | 282 | | II.4 | human | stool | 2009 | 2006 | 12 |
| 682 | Hong Kong | HM191761 | 109 | II.4 | | human | stool | 2010 | 2009 | unpublished |
| 683 | Hong Kong | HM191763 | 109 | II.4 | | human | stool | 2010 | 2009 | unpublished |
| 684 | Hong Kong | HM191764 | 109 | II.4 | | human | stool | 2010 | 2009 | unpublished |

| | | | | | | | | | | |
|---|---|---|---|---|---|---|---|---|---|---|
| 685 | Hong Kong | HM191765 | 461 | | II.4 | human | stool | 2010 | 2009 | unpublished |
| 686 | Hong Kong | HM191766 | 461 | | II.4 | human | stool | 2010 | 2009 | unpublished |
| 687 | Hong Kong | HM191767 | 461 | | II.4 | human | stool | 2010 | 2009 | unpublished |
| 688 | Hong Kong | HM191768 | 461 | | II.4 | human | stool | 2010 | 2009 | unpublished |
| 689 | Hong Kong | HM191769 | 1078 | II.4 | II.4 | human | stool | 2010 | 2009 | unpublished |
| 690 | Hong Kong | HM191770 | 1078 | II.4 | II.4 | human | stool | 2010 | 2009 | unpublished |
| 691 | Hong Kong | HM191771 | 1078 | II.4 | II.4 | human | stool | 2010 | 2009 | unpublished |
| 692 | Hong Kong | HM191772 | 1078 | II.4 | II.4 | human | stool | 2010 | 2009 | unpublished |
| 693 | Hong Kong | HM191773 | 2240 | II.4 | II.4 | human | stool | 2010 | 2009 | unpublished |
| 694 | Hong Kong | HM802525 | 3990 | II.4 | II.4 | human | stool | 2010 | 2006 | 14 |
| 695 | Hong Kong | HM802526 | 3990 | II.4 | II.4 | human | stool | 2010 | 2006 | 14 |
| 696 | Hong Kong | HM802527 | 3990 | II.4 | II.4 | human | stool | 2010 | 2006 | 14 |
| 697 | Hong Kong | HM802528 | 3989 | II.4 | II.4 | human | stool | 2010 | 2006 | 14 |
| 698 | Hong Kong | HM802529 | 3989 | II.4 | II.4 | human | stool | 2010 | 2006 | 14 |
| 699 | Hong Kong | HM802530 | 3989 | II.4 | II.4 | human | stool | 2010 | 2006 | 14 |
| 700 | Hong Kong | HM802531 | 3989 | II.4 | II.4 | human | stool | 2010 | 2006 | 14 |
| 701 | Hong Kong | HM802532 | 3989 | II.4 | II.4 | human | stool | 2010 | 2006 | 14 |
| 702 | Hong Kong | HM802533 | 3989 | II.4 | II.4 | human | stool | 2010 | 2006 | 14 |
| 703 | Hong Kong | HM802534 | 3989 | II.4 | II.4 | human | stool | 2010 | 2006 | 14 |
| 704 | Hong Kong | HM802535 | 3989 | II.4 | II.4 | human | stool | 2010 | 2006 | 14 |
| 705 | Hong Kong | HM802536 | 3989 | II.4 | II.4 | human | stool | 2010 | 2006 | 14 |
| 706 | Hong Kong | HM802537 | 3989 | II.4 | II.4 | human | stool | 2010 | 2006 | 14 |
| 707 | Hong Kong | HM802538 | 3989 | II.4 | II.4 | human | stool | 2010 | 2006 | 14 |
| 708 | Hong Kong | HM802539 | 3989 | II.4 | II.4 | human | stool | 2010 | 2006 | 14 |
| 709 | Hong Kong | HM802540 | 3989 | II.4 | II.4 | human | stool | 2010 | 2005 | 14 |
| 710 | Hong Kong | HM802541 | 3989 | II.4 | II.4 | human | stool | 2010 | 2004 | 14 |
| 711 | Hong Kong | HM802542 | 3989 | II.4 | II.4 | human | stool | 2010 | 2005 | 14 |
| 712 | Hong Kong | HM802543 | 3989 | II.4 | II.4 | human | stool | 2010 | 2005 | 14 |
| 713 | Hong Kong | HM802544 | 3989 | II.4 | II.4 | human | stool | 2010 | 2005 | 14 |
| 714 | Hong Kong | HM802545 | 3988 | II.4 | II.4 | human | stool | 2010 | 2005 | 14 |
| 715 | Hong Kong | HM802546 | 3988 | II.12 | II.4 | human | stool | 2010 | 2004 | 14 |
| 716 | Hong Kong | HM802547 | 3988 | II.12 | II.4 | human | stool | 2010 | 2004 | 14 |
| 717 | Hong Kong | HM802548 | 3988 | II.12 | II.4 | human | stool | 2010 | 2005 | 14 |
| 718 | Hong Kong | HM802549 | 3988 | II.12 | II.4 | human | stool | 2010 | 2005 | 14 |
| 719 | Hong Kong | HM802550 | 3988 | II.12 | II.4 | human | stool | 2010 | 2005 | 14 |
| 720 | Hong Kong | HM802551 | 3988 | II.12 | II.4 | human | stool | 2010 | 2004 | 14 |
| 721 | Hong Kong | HM802552 | 3988 | II.12 | II.4 | human | stool | 2010 | 2005 | 14 |
| 722 | Hong Kong | HM802553 | 3988 | II.12 | II.4 | human | stool | 2010 | 2005 | 14 |
| 723 | Hong Kong | HM802554 | 3988 | II.12 | II.4 | human | stool | 2010 | 2005 | 14 |
| 724 | Hong Kong | HM802555 | 3988 | II.12 | II.4 | human | stool | 2010 | 2005 | 14 |
| 725 | Hong Kong | HQ005292 | 2240 | II.4 | II.4 | human | stool | 2010 | 2009 | unpublished |
| 726 | Hong Kong | HQ005293 | 2240 | II.4 | II.4 | human | stool | 2010 | 2009 | unpublished |
| 727 | Hong Kong | HQ005294 | 2240 | II.4 | II.4 | human | stool | 2010 | 2009 | unpublished |

| | | | | | | | | | | |
|---|---|---|---|---|---|---|---|---|---|---|
| 728 | Hong Kong | HQ005295 | 2240 | II.4 | II.4 | human | stool | 2010 | 2009 | unpublished |
| 729 | Hong Kong | HQ005296 | 2240 | II.4 | II.4 | human | stool | 2010 | 2009 | unpublished |
| 730 | Hong Kong | HQ005297 | 2240 | II.4 | II.4 | human | stool | 2010 | 2009 | unpublished |
| 731 | Hong Kong | HQ005298 | 2240 | II.4 | II.4 | human | stool | 2010 | 2009 | unpublished |
| 732 | Hong Kong | HQ005299 | 2240 | II.4 | II.4 | human | stool | 2010 | 2010 | unpublished |
| 733 | Jiangsu | EU072213 | 274 | II.12 | | human | unclear | 2007 | 1999~2005 | unpublished |
| 734 | Jiangsu | EU072214 | 274 | II.12 | | human | unclear | 2007 | 1999~2005 | unpublished |
| 735 | Jiangsu | EU072216 | 274 | II.12 | | human | unclear | 2007 | 1999~2005 | unpublished |
| 736 | Jiangsu | EU072217 | 274 | II.12 | | human | unclear | 2007 | 1999~2005 | unpublished |
| 737 | Jiangsu | EU072221 | 274 | II.12 | | human | unclear | 2007 | 1999~2005 | unpublished |
| 738 | Jiangsu | EU072222 | 274 | II.12 | | human | unclear | 2007 | 1999~2005 | unpublished |
| 739 | Jiangsu | EU074212 | 274 | I.b | | human | unclear | 2007 | 1999~2005 | unpublished |
| 740 | Jiangsu | EU074213 | 274 | II.4 | | human | unclear | 2007 | 1999~2005 | unpublished |
| 741 | Jiangsu | EU074214 | 274 | II.4 | | human | unclear | 2007 | 1999~2005 | unpublished |
| 742 | Jiangsu | EU074215 | 274 | II.4 | | human | unclear | 2007 | 1999~2005 | unpublished |
| 743 | Jiangsu | EU074216 | 274 | II.4 | | human | unclear | 2007 | 1999~2005 | unpublished |
| 744 | Jilin | EF670593 | 281 | | II.3 | human | stool | 2007 | 2006 | 1 |
| 745 | Jilin | EF670594 | 292 | | I.2 | human | stool | 2007 | 2006 | 1 |
| 746 | Jilin | EF670595 | 291 | | I.2 | human | stool | 2007 | 2006 | 1 |
| 747 | Jilin | EF670596 | 292 | | I.2 | human | stool | 2007 | 2006 | 1 |
| 748 | Jilin | EF670597 | 292 | | I.2 | human | stool | 2007 | 2006 | 1 |
| 749 | Jilin | EF670604 | 281 | | II.4 | human | stool | 2007 | 2006 | 1 |
| 750 | Jilin | EF670605 | 281 | | II.4 | human | stool | 2007 | 2006 | 1 |
| 751 | Jilin | EF670606 | 281 | | II.4 | human | stool | 2007 | 2006 | 1 |
| 752 | Jilin | EF670607 | 281 | | II.4 | human | stool | 2007 | 2006 | 1 |
| 753 | Jilin | EF670636 | 281 | | II.4 | human | stool | 2007 | 2006 | 1 |
| 754 | Jilin | EF670637 | 281 | | II.4 | human | stool | 2007 | 2006 | 1 |
| 755 | Jilin | EF670638 | 281 | | II.4 | human | stool | 2007 | 2006 | 1 |
| 756 | Jilin | EF670639 | 281 | | II.4 | human | stool | 2007 | 2006 | 1 |
| 757 | Jilin | EF670640 | 281 | | II.4 | human | stool | 2007 | 2006 | 1 |
| 758 | Jilin | EU072241 | 3227 | II.b | II.3 | human | unclear | 2007 | 1999~2005 | unpublished |
| 759 | Jilin | EU072242 | 274 | II.4 | | human | unclear | 2007 | 1999~2005 | unpublished |
| 760 | Jilin | EU072243 | 3230 | II.b | II.3 | human | unclear | 2007 | 1999~2005 | unpublished |
| 761 | Jilin | EU072244 | 274 | II.12 | | human | unclear | 2007 | 1999~2005 | unpublished |
| 762 | Jilin | EU072246 | 274 | II.12 | | human | unclear | 2007 | 1999~2005 | unpublished |
| 763 | Jilin | EU072247 | 274 | II.12 | | human | unclear | 2007 | 1999~2005 | unpublished |
| 764 | Jilin | EU072248 | 274 | II.12 | | human | unclear | 2007 | 1999~2005 | unpublished |
| 765 | Jilin | EU072321 | 275 | II.4 | | human | unclear | 2007 | 1999~2005 | unpublished |
| 766 | Jilin | EU072322 | 275 | II.4 | | human | unclear | 2007 | 1999~2005 | unpublished |
| 767 | Jilin | EU072323 | 275 | II.4 | | human | unclear | 2007 | 1999~2005 | unpublished |
| 768 | Jilin | EU072324 | 275 | II.4 | | human | unclear | 2007 | 1999~2005 | unpublished |
| 769 | Jilin | EU072325 | 275 | II.4 | | human | unclear | 2007 | 1999~2005 | unpublished |
| 770 | Jilin | EU072326 | 275 | II.4 | | human | unclear | 2007 | 1999~2005 | unpublished |

| | | | | | | | | | | |
|---|---|---|---|---|---|---|---|---|---|---|
| 771 | Jilin | EU072327 | 275 | II.4 | | human | unclear | 2007 | 1999~2005 | unpublished |
| 772 | Jilin | EU072328 | 275 | II.4 | | human | unclear | 2007 | 1999~2005 | unpublished |
| 773 | Jilin | EU072329 | 274 | II.4 | | human | unclear | 2007 | 1999~2005 | unpublished |
| 774 | Jilin | EU072330 | 274 | I.b | | human | unclear | 2007 | 1999~2005 | unpublished |
| 775 | Jilin | EU072331 | 275 | II.4 | | human | unclear | 2007 | 1999~2005 | unpublished |
| 776 | Jilin | EU072332 | 274 | II.4 | | human | unclear | 2007 | 1999~2005 | unpublished |
| 777 | Jilin | EU072333 | 275 | II.4 | | human | unclear | 2007 | 1999~2005 | unpublished |
| 778 | Jilin | EU072334 | 275 | II.3 | | human | unclear | 2007 | 1999~2005 | unpublished |
| 779 | Jilin | EU072336 | 275 | II.4 | | human | unclear | 2007 | 1999~2005 | unpublished |
| 780 | Jilin | EU072337 | 275 | II.4 | | human | unclear | 2007 | 1999~2005 | unpublished |
| 781 | Jilin | EU400333 | 281 | | II.4 | human | stool | 2008 | 2006 | 1 |
| 782 | Jilin | EU400335 | 281 | | II.4 | human | stool | 2008 | 2006 | 1 |
| 783 | Jilin | EU400336 | 281 | | II.4 | human | stool | 2008 | 2006 | 1 |
| 784 | Jilin | EU400337 | 281 | | II.4 | human | stool | 2008 | 2006 | 1 |
| 785 | Jilin | EU400338 | 281 | | II.4 | human | stool | 2008 | 2006 | 1 |
| 786 | Jilin | EU400339 | 281 | | II.4 | human | stool | 2008 | 2006 | 1 |
| 787 | Jilin | EU400340 | 281 | | II.4 | human | stool | 2008 | 2006 | 1 |
| 788 | Jilin | EU400341 | 281 | | II.4 | human | stool | 2008 | 2006 | 1 |
| 789 | Jilin | EU400342 | 281 | | II.4 | human | stool | 2008 | 2006 | 1 |
| 790 | Jilin | EU400343 | 281 | | II.4 | human | stool | 2008 | 2006 | 1 |
| 791 | Jilin | EU400344 | 281 | | II.4 | human | stool | 2008 | 2006 | 1 |
| 792 | Jilin | EU400345 | 281 | | II.4 | human | stool | 2008 | 2006 | 1 |
| 793 | Jilin | EU400346 | 281 | | II.4 | human | stool | 2008 | 2006 | 1 |
| 794 | Jilin | EU400347 | 281 | | II.4 | human | stool | 2008 | 2006 | 1 |
| 795 | Jilin | EU400348 | 281 | | II.4 | human | stool | 2008 | 2006 | 1 |
| 796 | Jilin | EU400349 | 281 | | II.4 | human | stool | 2008 | 2006 | 1 |
| 797 | Jilin | EU400350 | 281 | | II.4 | human | stool | 2008 | 2006 | 1 |
| 798 | Jilin | EU400351 | 281 | | II.4 | human | stool | 2008 | 2006 | 1 |
| 799 | Jilin | EU400352 | 281 | | II.4 | human | stool | 2008 | 2006 | 1 |
| 800 | Jilin | EU400353 | 281 | | II.4 | human | stool | 2008 | 2006 | 1 |
| 801 | Jilin | EU400354 | 281 | | II.4 | human | stool | 2008 | 2006 | 1 |
| 802 | Jilin | EU400355 | 281 | | II.4 | human | stool | 2008 | 2006 | 1 |
| 803 | Jilin | EU400356 | 281 | | II.4 | human | stool | 2008 | 2006 | 1 |
| 804 | Jilin | EU400357 | 281 | | II.4 | human | stool | 2008 | 2006 | 1 |
| 805 | Jilin | EU400358 | 281 | | II.4 | human | stool | 2008 | 2006 | 1 |
| 806 | Jilin | EU400359 | 281 | | II.4 | human | stool | 2008 | 2006 | 1 |
| 807 | Jilin | EU400372 | 281 | | II.4 | human | stool | 2008 | 2006 | 1 |
| 808 | Jilin | EU400373 | 281 | | II.4 | human | stool | 2008 | 2006 | 1 |
| 809 | Jilin | EU400374 | 281 | | II.4 | human | stool | 2008 | 2006 | 1 |
| 810 | Jilin | EU400375 | 281 | | II.4 | human | stool | 2008 | 2006 | 1 |
| 811 | Jilin | EU400376 | 281 | | II.4 | human | stool | 2008 | 2006 | 1 |
| 812 | Jilin | EU400378 | 281 | | II.3 | human | stool | 2008 | 2006 | 1 |
| 813 | Jilin | EU400380 | 293 | | I.4 | human | stool | 2008 | 2006 | 1 |

| | | | | | | | | | | |
|---|---|---|---|---|---|---|---|---|---|---|
| 814 | Liaoning | GQ402329 | 474 | II.4 | | human | stool | 2009 | 2007 | 10 |
| 815 | Liaoning | GQ402330 | 474 | II.4 | | human | stool | 2009 | 2007 | 10 |
| 816 | Liaoning | GQ402331 | 474 | II.4 | | human | stool | 2009 | 2007 | 10 |
| 817 | Liaoning | GQ402332 | 474 | II.4 | | human | stool | 2009 | 2007 | 10 |
| 818 | Liaoning | GQ402333 | 474 | II.4 | | human | stool | 2009 | 2007 | 10 |
| 819 | Liaoning | GQ402334 | 474 | II.4 | | human | stool | 2009 | 2007 | 10 |
| 820 | Liaoning | GQ402335 | 474 | II.4 | | human | stool | 2009 | 2007 | 10 |
| 821 | Liaoning | GQ402336 | 474 | II.4 | | human | stool | 2009 | 2007 | 10 |
| 822 | Liaoning | GQ402337 | 474 | II.4 | | human | stool | 2009 | 2007 | 10 |
| 823 | Liaoning | GQ402338 | 474 | II.4 | | human | stool | 2009 | 2007 | 10 |
| 824 | Liaoning | GQ402339 | 474 | II.4 | | human | stool | 2009 | 2007 | 10 |
| 825 | Liaoning | GQ402340 | 474 | II.4 | | human | stool | 2009 | 2007 | 10 |
| 826 | Liaoning | GQ402341 | 474 | II.4 | | human | stool | 2009 | 2007 | 10 |
| 827 | Liaoning | GQ402342 | 474 | II.4 | | human | stool | 2009 | 2007 | 10 |
| 828 | Liaoning | GQ402343 | 474 | II.4 | | human | stool | 2009 | 2007 | 10 |
| 829 | Liaoning | GQ402344 | 474 | II.4 | | human | stool | 2009 | 2007 | 10 |
| 830 | Liaoning | GQ402345 | 474 | II.4 | | human | stool | 2009 | 2007 | 10 |
| 831 | Liaoning | GQ402346 | 474 | II.4 | | human | stool | 2009 | 2007 | 10 |
| 832 | Liaoning | GQ402347 | 474 | II.4 | | human | stool | 2009 | 2007 | 10 |
| 833 | Liaoning | GQ402348 | 474 | II.4 | | human | stool | 2009 | 2007 | 10 |
| 834 | Liaoning | GQ402349 | 474 | II.4 | | human | stool | 2009 | 2007 | 10 |
| 835 | Shaanxi | EF670610 | 281 | | II.4 | human | stool | 2007 | 2006 | 1 |
| 836 | Shaanxi | EF670611 | 281 | | II.3 | human | stool | 2007 | 2006 | 1 |
| 837 | Shaanxi | EF670612 | 281 | | II.4 | human | stool | 2007 | 2006 | 1 |
| 838 | Shaanxi | EF670613 | 281 | | II.4 | human | stool | 2007 | 2006 | 1 |
| 839 | Shaanxi | EF670614 | 281 | | II.3 | human | stool | 2007 | 2006 | 1 |
| 840 | Shaanxi | EF670615 | 281 | | II.4 | human | stool | 2007 | 2006 | 1 |
| 841 | Shaanxi | EF670616 | 281 | | II.4 | human | stool | 2007 | 2006 | 1 |
| 842 | Shaanxi | EF670617 | 281 | | II.4 | human | stool | 2007 | 2006 | 1 |
| 843 | Shaanxi | EF670618 | 281 | | II.4 | human | stool | 2007 | 2006 | 1 |
| 844 | Shaanxi | EF670619 | 281 | | II.4 | human | stool | 2007 | 2006 | 1 |
| 845 | Shaanxi | EF670620 | 281 | | II.4 | human | stool | 2007 | 2006 | 1 |
| 846 | Shaanxi | EF670621 | 281 | | II.4 | human | stool | 2007 | 2006 | 1 |
| 847 | Shandong | DQ354238 | 274 | II.12 | | human | stool | 2006 | 2004-2005 | 4 |
| 848 | Shandong | DQ354239 | 274 | II.12 | | human | stool | 2006 | 2004-2005 | 4 |
| 849 | Shandong | DQ354240 | 274 | II.12 | | human | stool | 2006 | 2004-2005 | 4 |
| 850 | Shandong | DQ354241 | 274 | II.12 | | human | stool | 2006 | 2004-2005 | 4 |
| 851 | Shandong | DQ354242 | 274 | II.12 | | human | stool | 2006 | 2004-2005 | 4 |
| 852 | Shandong | DQ354243 | 274 | II.12 | | human | stool | 2006 | 2004-2005 | 4 |
| 853 | Shandong | DQ354244 | 274 | II.12 | | human | stool | 2006 | 2004-2005 | 4 |
| 854 | Shandong | DQ354245 | 274 | II.12 | | human | stool | 2006 | 2004-2005 | 4 |
| 855 | Shandong | DQ354246 | 274 | II.12 | | human | stool | 2006 | 2004-2005 | 4 |
| 856 | Shandong | DQ354247 | 274 | II.4 | | human | stool | 2006 | 2004-2005 | 4 |

| | | | | | | | | | | |
|---|---|---|---|---|---|---|---|---|---|---|
| 857 | Shandong | DQ354248 | 274 | II.4 | | human | stool | 2006 | 2004-2005 | 4 |
| 858 | Shandong | DQ354249 | 274 | II.4 | | human | stool | 2006 | 2004-2005 | 4 |
| 859 | Shandong | DQ354250 | 274 | II.4 | | human | stool | 2006 | 2004-2005 | 4 |
| 860 | Shandong | DQ354251 | 274 | II.4 | | human | stool | 2006 | 2004-2005 | 4 |
| 861 | Shandong | DQ354252 | 274 | II.4 | | human | stool | 2006 | 2004-2005 | 4 |
| 862 | Shandong | DQ354253 | 274 | II.4 | | human | stool | 2006 | 2004-2005 | 4 |
| 863 | Shandong | DQ354254 | 274 | II.4 | | human | stool | 2006 | 2004-2005 | 4 |
| 864 | Shandong | DQ354255 | 274 | II.b | | human | stool | 2006 | 2004-2005 | 4 |
| 865 | Shandong | DQ354256 | 274 | II.b | | human | stool | 2006 | 2004-2005 | 4 |
| 866 | Shandong | DQ354257 | 274 | II.b | | human | stool | 2006 | 2004-2005 | 4 |
| 867 | Shandong | DQ354258 | 274 | II.b | | human | stool | 2006 | 2004-2005 | 4 |
| 868 | Shandong | DQ354259 | 274 | II.b | | human | stool | 2006 | 2004-2005 | 4 |
| 869 | Shanghai | EF670627 | 281 | | II.4 | human | stool | 2007 | 2006 | 1 |
| 870 | Shanghai | EF670628 | 281 | | II.4 | human | stool | 2007 | 2006 | 1 |
| 871 | Shanghai | EF670629 | 281 | | II.4 | human | stool | 2007 | 2006 | 1 |
| 872 | Shanghai | EU072204 | 274 | II.12 | | human | unclear | 2007 | 1999~2005 | unpublished |
| 873 | Shanghai | EU072205 | 274 | II.12 | | human | unclear | 2007 | 1999~2005 | unpublished |
| 874 | Shanghai | EU072208 | 274 | II.12 | | human | unclear | 2007 | 1999~2005 | unpublished |
| 875 | Shanghai | EU072209 | 274 | II.12 | | human | unclear | 2007 | 1999~2005 | unpublished |
| 876 | Shanghai | EU072223 | 274 | II.12 | | human | unclear | 2007 | 1999~2005 | unpublished |
| 877 | Shanghai | EU072224 | 274 | II.12 | | human | unclear | 2007 | 1999~2005 | unpublished |
| 878 | Shanghai | EU072225 | 274 | II.12 | | human | unclear | 2007 | 1999~2005 | unpublished |
| 879 | Shanghai | EU072226 | 274 | II.12 | | human | unclear | 2007 | 1999~2005 | unpublished |
| 880 | Shanghai | EU072236 | 274 | II.3 | | human | unclear | 2007 | 1999~2005 | unpublished |
| 881 | Shanghai | EU072237 | 274 | II.12 | | human | unclear | 2007 | 1999~2005 | unpublished |
| 882 | Shanghai | EU400369 | 281 | | II.4 | human | stool | 2008 | 2006 | 1 |
| 883 | Shanghai | EU400370 | 281 | | II.4 | human | stool | 2008 | 2006 | 1 |
| 884 | Shanghai | EU400371 | 281 | | II.4 | human | stool | 2008 | 2006 | 1 |
| 885 | Shanghai | EU400379 | 281 | | II.3 | human | stool | 2008 | 2006 | 1 |
| 886 | Shanghai | GU991353 | 7555 | II.4 | II.4 | human | stool | 2010 | 2008 | 13 |
| 887 | Shanghai | GU991354 | 7511 | II.4 | II.4 | human | stool | 2010 | 2008 | 13 |
| 888 | Shanghai | GU991355 | 7544 | II.12 | II.3 | human | stool | 2010 | 2008 | 13 |
| 889 | Shanghai | JN596792 | 282 | | II.12 | human | stool | 2011 | 2009 | 16 |
| 890 | Shanghai | JN596795 | 282 | | II.2 | human | stool | 2011 | 2009 | 16 |
| 891 | Shanghai | JN596800 | 282 | | II.4 | human | stool | 2011 | 2008 | 16 |
| 892 | Shanghai | JN596802 | 282 | | II.4 | human | stool | 2011 | 2009 | 16 |
| 893 | Shanghai | JN596806 | 282 | | II.6 | human | stool | 2011 | 2008 | 16 |
| 894 | Shanghai | JN596810 | 282 | | II.14 | human | stool | 2011 | 2009 | 16 |
| 895 | Shanghai | JN596811 | 282 | | II.3 | human | stool | 2011 | 2009 | 16 |
| 896 | Shanxi | EF670622 | 281 | | II.4 | human | stool | 2007 | 2006 | 1 |
| 897 | Shanxi | EF670623 | 281 | | II.3 | human | stool | 2007 | 2006 | 1 |
| 898 | Shanxi | EF670650 | 7359 | II.7 | II.14 | human | stool | 2007 | 2006 | 1 |
| 899 | Shanxi | EU400331 | 281 | | II.4 | human | stool | 2008 | 2006 | 1 |

| | | | | | | | | | | |
|---|---|---|---|---|---|---|---|---|---|---|
| 900 | Shanxi | EU400332 | 281 | | II.4 | human | stool | 2008 | 2006 | 1 |
| 901 | Sichuan | HM624049 | 387 | | II.4 | human | stool | 2010 | 2010 | unpublished |
| 902 | Tianjin | GQ379132 | 346 | | II.4 | human | stool | 2009 | 2008 | 7 |
| 903 | Tianjin | GQ379133 | 346 | | II.4 | human | stool | 2009 | 2008 | 7 |
| 904 | Tianjin | GQ379134 | 346 | | II.4 | human | stool | 2009 | 2008 | 7 |
| 905 | Tianjin | GQ379135 | 346 | | II.4 | human | stool | 2009 | 2008 | 7 |
| 906 | Tianjin | GQ379136 | 346 | | II.4 | human | stool | 2009 | 2008 | 7 |
| 907 | Tianjin | GQ379137 | 346 | | II.4 | human | stool | 2009 | 2008 | 7 |
| 908 | Tianjin | GQ379138 | 346 | | II.4 | human | stool | 2009 | 2008 | 7 |
| 909 | Tianjin | GQ379139 | 346 | | II.4 | human | stool | 2009 | 2008 | 7 |
| 910 | Tianjin | GQ379140 | 346 | | II.4 | human | stool | 2009 | 2008 | 7 |
| 911 | Tianjin | GQ379141 | 346 | | II.4 | human | stool | 2009 | 2008 | 7 |
| 912 | Tianjin | GQ379142 | 346 | | II.4 | human | stool | 2009 | 2008 | 7 |
| 913 | Tianjin | GQ379143 | 346 | | II.4 | human | stool | 2009 | 2008 | 7 |
| 914 | Tianjin | GQ379144 | 346 | | II.4 | human | stool | 2009 | 2008 | 7 |
| 915 | Tianjin | GQ379145 | 346 | | II.4 | human | stool | 2009 | 2008 | 7 |
| 916 | Tianjin | GQ379146 | 346 | | II.3 | human | stool | 2009 | 2008 | 7 |
| 917 | Tianjin | GQ379147 | 346 | | II.4 | human | stool | 2009 | 2008 | 7 |
| 918 | Tianjin | GQ379148 | 346 | | II.4 | human | stool | 2009 | 2008 | 7 |
| 919 | Tianjin | GQ379149 | 346 | | II.4 | human | stool | 2009 | 2008 | 7 |
| 920 | Tianjin | GQ379150 | 346 | | II.4 | human | stool | 2009 | 2008 | 7 |
| 921 | Tianjin | GQ379151 | 346 | | II.4 | human | stool | 2009 | 2008 | 7 |
| 922 | Tianjin | GQ379152 | 346 | | II.4 | human | stool | 2009 | 2008 | 7 |
| 923 | Tianjin | GQ379153 | 346 | | II.4 | human | stool | 2009 | 2008 | 7 |
| 924 | Tianjin | GQ379154 | 346 | | II.4 | human | stool | 2009 | 2008 | 7 |
| 925 | Tianjin | GQ379155 | 346 | | II.3 | human | stool | 2009 | 2009 | 7 |
| 926 | Tianjin | JN596790 | 282 | | II.12 | human | stool | 2011 | 2009 | 16 |
| 927 | Tianjin | JN596793 | 282 | | II.16 | human | stool | 2011 | 2008 | 16 |
| 928 | Tianjin | JN596797 | 282 | | II.13 | human | stool | 2011 | 2008 | 16 |
| 929 | Tianjin | JN596801 | 282 | | II.4 | human | stool | 2011 | 2008 | 16 |
| 930 | Tianjin | JN596805 | 282 | | II.4 | human | stool | 2011 | 2009 | 16 |
| 931 | Tianjin | JN596809 | 282 | | II.6 | human | stool | 2011 | 2009 | 16 |
| 932 | Tianjin | JN596812 | 282 | | II.3 | human | stool | 2011 | 2009 | 16 |
| 933 | Xinjiang | EU072210 | 274 | II.12 | | human | unclear | 2007 | 1999~2005 | unpublished |
| 934 | Xinjiang | EU072211 | 274 | II.12 | | human | unclear | 2007 | 1999~2005 | unpublished |
| 935 | Xinjiang | EU072212 | 273 | II.12 | | human | unclear | 2007 | 1999~2005 | unpublished |
| 936 | Xinjiang | EU072218 | 274 | II.12 | | human | unclear | 2007 | 1999~2005 | unpublished |
| 937 | Xinjiang | EU072230 | 274 | II.12 | | human | unclear | 2007 | 1999~2005 | unpublished |
| 938 | Yunnan | DQ304651 | 1024 | II.7 | II.7 | human | stool | 2005 | 2004 | 3 |
| 939 | Yunnan | EU072202 | 274 | II.8 | | human | unclear | 2007 | 1999~2005 | unpublished |
| 940 | Yunnan | EU072203 | 274 | II.12 | | human | unclear | 2007 | 1999~2005 | unpublished |
| 941 | Yunnan | EU072206 | 274 | II.12 | | human | unclear | 2007 | 1999~2005 | unpublished |
| 942 | Yunnan | EU072207 | 274 | II.12 | | human | unclear | 2007 | 1999~2005 | unpublished |

| | | | | | | | | | | |
|---|---|---|---|---|---|---|---|---|---|---|
| 943 | Yunnan | EU072238 | 274 | II.3 | | human | unclear | 2007 | 1999~2005 | unpublished |
| 944 | Yunnan | EU072239 | 274 | II.12 | | human | unclear | 2007 | 1999~2005 | unpublished |
| 945 | Yunnan | EU072240 | 275 | II.12 | | human | unclear | 2007 | 1999~2005 | unpublished |
| 946 | Yunnan | EU072278 | 274 | II.4 | | human | unclear | 2007 | 1999~2005 | unpublished |
| 947 | Yunnan | EU072282 | 274 | II.4 | | human | unclear | 2007 | 1999~2005 | unpublished |
| 948 | Yunnan | EU072283 | 274 | II.3 | | human | unclear | 2007 | 1999~2005 | unpublished |
| 949 | Yunnan | EU072284 | 274 | II.3 | | human | unclear | 2007 | 1999~2005 | unpublished |
| 950 | Yunnan | EU072285 | 274 | II.4 | | human | unclear | 2007 | 1999~2005 | unpublished |
| 951 | Yunnan | EU072300 | 274 | II.7 | | human | unclear | 2007 | 1999~2005 | unpublished |
| 952 | Yunnan | EU072340 | 274 | II.4 | | human | unclear | 2007 | 1999~2005 | unpublished |
| 953 | Yunnan | EU072344 | 274 | II.12 | | human | unclear | 2007 | 1999~2005 | unpublished |
| 954 | Yunnan | EU072345 | 274 | II.12 | | human | unclear | 2007 | 1999~2005 | unpublished |
| 955 | Yunnan | EU072346 | 274 | II.3 | | human | unclear | 2007 | 1999~2005 | unpublished |
| 956 | Zhejiang | EU072287 | 274 | II.a | | human | unclear | 2007 | 1999~2005 | unpublished |
| 957 | Zhejiang | EU072288 | 274 | II.4 | | human | unclear | 2007 | 1999~2005 | unpublished |
| 958 | Zhejiang | EU072289 | 274 | II.a | | human | unclear | 2007 | 1999~2005 | unpublished |
| 959 | Zhejiang | EU072290 | 274 | II.4 | | human | unclear | 2007 | 1999~2005 | unpublished |
| 960 | Zhejiang | EU072291 | 274 | II.4 | | human | unclear | 2007 | 1999~2005 | unpublished |
| 961 | Zhejiang | EU072292 | 274 | II.4 | | human | unclear | 2007 | 1999~2005 | unpublished |
| 962 | Zhejiang | EU072293 | 274 | II.a | | human | unclear | 2007 | 1999~2005 | unpublished |
| 963 | Zhejiang | EU072294 | 274 | II.a | | human | unclear | 2007 | 1999~2005 | unpublished |
| 964 | Zhejiang | EU072295 | 274 | II.4 | | human | unclear | 2007 | 1999~2005 | unpublished |
| 965 | Zhejiang | EU072296 | 274 | II.4 | | human | unclear | 2007 | 1999~2005 | unpublished |
| 966 | Zhejiang | EU072297 | 274 | II.4 | | human | unclear | 2007 | 1999~2005 | unpublished |
| 967 | Zhejiang | EU072298 | 274 | II.4 | | human | unclear | 2007 | 1999~2005 | unpublished |
| 968 | Zhejiang | HQ680712 | 270 | I.b | | human | unclear | 2010 | 2008 | unpublished |
| 969 | Zhejiang | HQ680713 | 266 | I.b | | human | unclear | 2010 | 2008 | unpublished |
| 970 | Zhejiang | HQ680714 | 260 | I.8 | | human | unclear | 2010 | 2009 | unpublished |
| 971 | Zhejiang | HQ680715 | 270 | I.8 | | human | unclear | 2010 | 2008 | unpublished |
| 972 | Zhejiang | HQ680716 | 270 | I.8 | | human | unclear | 2010 | 2008 | unpublished |
| 973 | Zhejiang | HQ680717 | 270 | II.4 | | human | unclear | 2010 | 2008 | unpublished |
| 974 | Zhejiang | HQ680718 | 270 | II.4 | | human | unclear | 2010 | 2008 | unpublished |
| 975 | Zhejiang | HQ680719 | 270 | II.4 | | human | unclear | 2010 | 2009 | unpublished |
| 976 | Zhejiang | HQ680720 | 255 | II.b | | human | unclear | 2010 | 2009 | unpublished |
| 977 | Zhejiang | HQ680721 | 270 | II.4 | | human | unclear | 2010 | 2009 | unpublished |
| 978 | Zhejiang | HQ840433 | 285 | II.4 | | human | unclear | 2010 | 2010 | unpublished |
| 979 | Zhejiang | HQ840434 | 285 | II.4 | | human | unclear | 2010 | 2010 | unpublished |
| 980 | Zhejiang | JN596799 | 282 | | II.4 | human | stool | 2011 | 2008 | 16 |
| 981 | Zhejiang | JN596803 | 282 | | II.4 | human | stool | 2011 | 2009 | 16 |
| 982 | Zhejiang | JN596808 | 282 | | II.6 | human | stool | 2011 | 2008 | 16 |
| 983 | Zhejiang | JN596814 | 282 | | II.3 | human | stool | 2011 | 2009 | 16 |

**References**


1. Jin M, Xie H, Duan Z, et al. Emergence of the GII4/2006b variant and recombinant noroviruses in China. Journal of medical virology, 2008, 80(11): 1997-2004.
2. Ho E, Cheng P K C, Wong D A, et al. Correlation of norovirus variants with epidemics of acute viral gastroenteritis in Hong Kong. Journal of medical virology, 2006, 78(11): 1473-1479.
3. Phan T G, Yan H, Li Y, et al. Novel recombinant norovirus in China. Emerging Infectious Diseases, 2006, 12(5): 857.
4. Si H L, et al. Characterization of human calicivirus prevalence in infants with acute diarrhea in Jinan area, China. Chinese Journal of Pathogen Biology, 2006.1(2): 1673-5234.
5. Guo L, Song J, Xu X, et al. Genetic analysis of norovirus in children affected with acute gastroenteritis in Beijing, 2004–2007. Journal of Clinical Virology, 2009, 44(1): 94-98.
6. He Y Q, Ma H W, Yao X J, et al. Norovirus gastroenteritis outbreak is associated with contaminated drinking water in South China. Food and Environmental Virology, 2010, 2(4): 207-210.
7. Ouyang Y, Ma H, Jin M, et al. Etiology and epidemiology of viral diarrhea in children under the age of five hospitalized in Tianjin, China. Archives of virology, 2012, 157(5): 881-887.
8. Liu L J, Liu W, Liu Y X, et al. Identification of norovirus as the top enteric viruses detected in adult cases with acute gastroenteritis. The American journal of tropical medicine and hygiene, 2010, 82(4): 717.
9. Li C S Y, Chan P K S, Tang J W. Prevalence of diarrhea viruses in hospitalized children in Hong Kong in 2008. Journal of medical virology, 2009, 81(11): 1903-1911.
10. Wang Z S, et al.. Detection of norovirus by reverse transcription polymerase chain reaction and its phylogenetic analysis. Chinese Journal of Health Laboratory Technology, 2008, 18(5): 783-785.
11. Jin M, Chen J, Zhang X, et al. Genetic diversity of noroviruses in Chinese adults: Potential recombination hotspots and GII-4/Den Haag-specific mutations at a putative epitope. Infection, Genetics and Evolution, 2011, 11(7): 1716-1726.
12. Chan M C W, Lee N, Wong R Y K, et al. A 75-year-old woman with seasonal influenza A (H3N2) virus infection and diarrhea. Journal of Clinical Virology, 2010, 48(4): 231-233.
13. Shen Q, Zhang W, Yang S, et al. Genomic organization and recombination analysis of human norovirus identified from China. Molecular biology reports, 2012,

39(2): 1275-1281.
14. Chan M C W, Lee N, Ho W S, et al. Covariation of major and minor viral capsid proteins in norovirus genogroup II genotype 4 strains. Journal of virology, 2012, 86(2): 1227-1232.
15. Gao Y, Jin M, Cong X, et al. Clinical and Molecular epidemiologic analyses of norovirus-associated sporadic gastroenteritis in adults from Beijing, China. Journal of medical virology, 2011, 83(6): 1078-1085.
16. Zeng M, Xu X, Zhu C, et al. Clinical and molecular epidemiology of norovirus infection in childhood diarrhea in China. Journal of medical virology, 2012, 84(1): 145-151.
